# Identification and Characterization of MicroRNAs Associated with Somatic Copy Number Alterations in Cancer

**DOI:** 10.3390/cancers10120475

**Published:** 2018-11-29

**Authors:** Jihee Soh, Hyejin Cho, Chan-Hun Choi, Hyunju Lee

**Affiliations:** 1 School of Electrical Engineering and Computer Science, Gwangju Institute of Science and Technology, Gwangju 61005, Korea; jhsoh@gist.ac.kr (J.S.); heyjin@gist.ac.kr (H.C.); 2 College of Korean Medicine, Dongshin University, Naju-si, Jeollanam-do 58245, Korea; mensolog@dsu.ac.kr

**Keywords:** MicroRNA, copy number alteration, Co-regulation, cancer

## Abstract

MicroRNAs (miRNAs) are key molecules that regulate biological processes such as cell proliferation, differentiation, and apoptosis in cancer. Somatic copy number alterations (SCNAs) are common genetic mutations that play essential roles in cancer development. Here, we investigated the association between miRNAs and SCNAs in cancer. We collected 2538 tumor samples for seven cancer types from The Cancer Genome Atlas. We found that 32−84% of miRNAs are in SCNA regions, with the rate depending on the cancer type. In these regions, we identified 80 SCNA-miRNAs whose expression was mainly associated with SCNAs in at least one cancer type and showed that these SCNA-miRNAs are related to cancer by survival analysis and literature searching. We also identified 58 SCNA-miRNAs common in the seven cancer types (CC-SCNA-miRNAs) and showed that these CC-SCNA-miRNAs are more likely to be related with protein and gene expression than other miRNAs. Furthermore, we experimentally validated the oncogenic role of miR-589. In conclusion, our results suggest that SCNA-miRNAs significantly alter biological processes related to cancer development, confirming the importance of SCNAs in non-coding regions in cancer.

## 1. Introduction

MicroRNAs (miRNAs) are small, single-stranded non-coding RNA molecules, 17–25 nucleotides long, that act as post-transcriptional regulators of gene expression by binding to complementary sequences in the 3’ untranslated region of target messenger RNAs (mRNAs) [1,2]. Recently, miRNAs have been investigated as key molecules involved in regulating important cancer-associated processes, such as cell proliferation, differentiation, and apoptosis [3]. Specific miRNAs were identified as biomarkers affecting the expression levels of genes involved in cancer development [4]. Hamilton et al. [4]. identified a pan-cancer oncogenic miRNA network by integrating Argonaute Crossing Immunoprecipitation datasets and The Cancer Genome Atlas (TCGA) datasets containing data for 12 cancer types and showed that members of a microRNA superfamily, including miR-17, miR-19, and miR-130, co-target critical tumor suppressors. Because of their important roles in cancer, it is important to understand the regulation of miRNA biogenesis. The regulation of miRNAs has been evaluated at different levels, including in studies of genetic mutations, epigenetic modifications, and alterations in the microprocessor components, such as DROSHA, DGCR8, and DICER1 [5].

Somatic copy number alterations (SCNAs) are common genetic alterations that affect cancer development. Zack et al. [6] analyzed high-resolution copy number profiles of 4934 cancer patients obtained from TCGA and found common SCNA patterns across 11 cancer types. Alterations in genetic regions containing oncogenes and tumor suppressor genes were observed, which were shown to affect transcriptional activation or inactivation associated with cancer development. Additionally, several studies detected a complex association between SCNAs and gene expression levels in cancer [7,8].

Although studies have investigated the relationship between miRNAs and SCNAs, most were focused on specific miRNAs, SCNAs of specific genes, or a single cancer type [9,10,11,12,13,14,15,16]. The genome-wide association between miRNA expression and SCNAs across several cancer types has not been widely investigated. Calin et al. [17] showed that 52.5% miRNAs are located in fragile sites (FRAs) or cancer-related genomic regions and suggested that miRNAs are more likely to be at FRAs than at non-FRAs. Furthermore, they showed that the expression of several miRNAs in deleted regions is down-regulated in B-cell chronic lymphocytic leukemias. Wilting et al. [18] showed that the differentially expressed miRNAs can be partially associated with chromosomal alterations in cervical cancer. Furthermore, Zhang et al. [19] investigated DNA copy number variable regions containing 283 known human miRNA genes involved in the development of ovarian cancer, breast cancer, and melanoma, and demonstrated that SCNAs are associated with altered miRNA expression. In contrast, Lamy et al. [20] found no significant relationship between the copy numbers and expression levels of 18 and 41 miRNAs differentially expressed in colon and prostate cancers, respectively. These inconsistencies may be related to the relatively small number of genes and samples used in the studies. With the availability of large-scale data sets such as TCGA, miRNA genomic instability and the relationship between copy number alterations and miRNA expression can be examined in greater detail.

In this study, we evaluated the association between SCNAs and miRNA expression and potential effects of SCNAs on miRNA-related oncogenesis. We collected the paired copy number and expression profiles of 971 miRNAs from 2538 tumor samples across seven cancer types from TCGA [21,22,23,24,25,26,27]: urothelial bladder carcinoma (BLCA), breast invasive carcinoma (BRCA), head and neck squamous cell carcinoma (HNSC), kidney renal clear cell carcinoma (KIRC), lung adenocarcinoma (LUAD), lung squamous cell carcinoma (LUSC), and uterine corpus endometrial carcinoma (UCEC). First, we investigated whether the genomic locations of miRNAs represent copy number-aberrant regions. Furthermore, we identified SCNA-associated miRNAs and evaluated their involvement oncogenesis. Additionally, we identified miRNAs associated with SCNAs across different cancer types and investigated whether these pan-cancer miRNAs are more likely to alter the expression levels of their target genes compared to other miRNAs.

## 2. Results

### 2.1. MiRNA Localization in the Genomic Regions with SCNAs in Cancers

**Figure 1:**
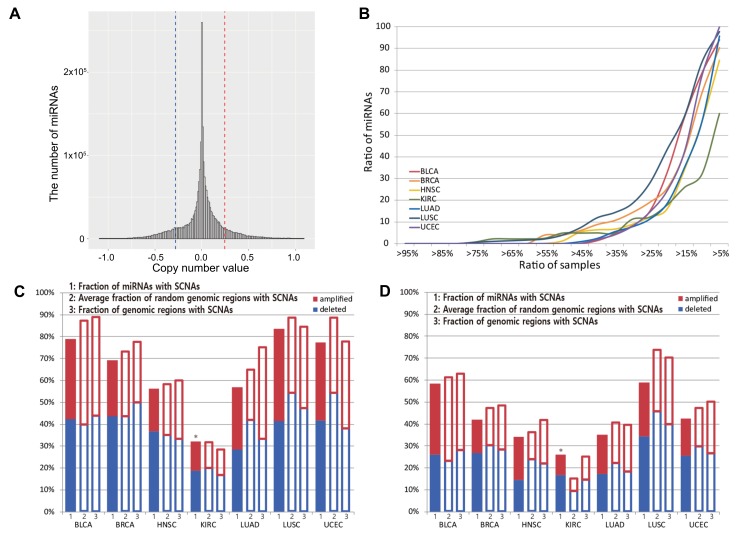
Characteristics of miRNAs with copy number alterations. (**A**) Copy number distribution of miRNA gene regions. Red and blue dashed lines show copy number amplification or deletion thresholds, respectively. (**B**) On the *x* axis, the fraction of samples containing somatic copy number alterations (SCNAs) is represented. On the *y* axis, the fraction of miRNAs with SCNAs is presented when an miRNA was declared to be located in the SCNA region if the fraction of samples with SCNAs was larger than the given fraction value on the *x* axis. (**C**) The ratio of miRNAs with SCNAs in >10% of samples of each cancer type. (**D**) The ratio of miRNAs with SCNAs in >15% of samples of each cancer type. In (**C**,**D**), red and blue bars represent the percentage of miRNAs with copy number amplifications or deletions, respectively. These ratios were compared to those of SCNAs in randomly selected regions and all genomic regions. “*” indicates that the fraction of miRNAs in the SCNA regions was significantly larger than the fraction of random genomic regions with SCNAs for the KIRC.

We obtained information on the genomic location of miRNA precursors from the miRBase [28] (v21, June 2014), and determined the log2 ratios of somatic copy numbers values using level 3 segmented copy number data from TCGA. Next, we drew copy number distributions of 971 miRNAs for all samples and defined the highest and lowest β% = 10% values as copy number amplification and deletion thresholds, which correspond to 0.25 and −0.28 log2 ratios, respectively (Figure 1A). When the log2 ratio of the copy number of an miRNA gene in a given sample was larger than 0.25 or smaller than −0.28, we considered that it was amplified or deleted, respectively, in the sample. To select copy number amplification and deletion thresholds, we converted 5%, 10%, and 15% relative copy number values into integer copy number values using the ABSOLUTE [29] algorithm by considering the ploidy and purity of the cancer samples. Ploidy and purity values of cancer samples were obtained from https://doi.org/10.7303/syn1710466.2, which were estimated by ABSOLUTE. For amplification, the integer copy number values corresponding to 5%, 10%, and 15% were 3.66, 3.07, and 2.78 copies, respectively. For deletion, the integer copy number values corresponding to 5%, 10%, and 15% were 1.47, 1.65, and 1.80 copies, respectively. Although ABSOLUTE is widely used for estimating the ploidy and purity of samples, this algorithm tends to overestimate ploidy [30]. Thus, in this study, we chose 10% values corresponding to 3.07 copies and 1.65 copies for amplification and deletion, respectively, after considering overestimation, although this threshold may be rather strict.

Next, we determined the number of miRNAs in the SCNA region of different cancer types. The location and amount of SCNAs differed between patients even in those with the same cancer types. Thus, we identified miRNA genes recurrently aberrant in copy numbers across multiple samples for a given cancer type. Figure 1B shows the number of miRNAs in SCNA regions across multiple samples. For example, when we counted miRNAs in SCNA regions in > 10% of KIRC samples, 32% of miRNAs were selected. Various fractions of samples in which miRNAs are in the SCNA regions (>5%, 10%, 15%, ... >95%) were examined. As expected, as the fraction of samples increased, the ratio of miRNAs decreased. Particularly, 32−84% and 26−59% of miRNAs were in the SCNA regions in >10% and >15% of tumor samples of most cancer types, respectively. As shown in Figure 1C,D, these miRNAs were selected for analysis. Note that in a study by Zhang et al. [19], miRNAs observed in >15% samples with SCNAs were considered significant.

The results presented in Figure 1C show that 78.68% (764/971) of miRNAs are in the SCNA regions in BLCA, 69.52% (675/971) in BRCA, 56.33% (547/971) in HNSC, 32.13% (312/971) in KIRC, 56.33% (547/971) in LUAD, 83.73% (813/971) in LUSC, and 77.55% (753/971) in UCEC samples when we considered miRNAs with SCNAs in >10% of tumor samples. Similarly, Figure 1D shows that 58.29% (566/971) of miRNAs are in the SCNA regions in BLCA, 41.92% (407/971) in BRCA, 34.19% (332/971) in HNSC, 25.95% (252/971) in KIRC, 35.12% (341/971) in LUAD, 58.81% (571/971) in LUSC, and 42.53% (413/971) in UCEC samples when we considered miRNAs with SCNAs in >15% of tumor samples. Based on these results, BLCA and LUSC contain the largest fractions of miRNAs in regions with the SCNAs, while KIRC showed the lowest ratio of miRNAs in the SCNA regions compared to those in other cancer types.

For each cancer type, we investigated whether miRNAs tend to be present in the SCNA regions because previous studies [17,18,19] showed that miRNAs are more likely to be found in copy number-altered regions. We randomly selected 971 genomic regions and determined the fraction of random genomic regions with copy number amplifications or deletions (>0.25 and <−0.28) in 10% and 15% of samples. We repeated this random selection *N* = 1000 times and calculated the average fraction of random genomic regions with SCNAs (Figure 1C,D). Except for in KIRC, the fractions of SCNAs in miRNA locations were not higher than the fraction of SCNA regions in randomly selected regions in cancer. To examine whether miRNAs are more likely to be in SCNA regions in cancer, we calculated *p*-values for the research hypothesis that the fraction of miRNAs in SCNA regions (fraction_miRNA) is larger than the fraction of random genomic regions with SCNAs (fraction_random) as follows:

$$p-\mathrm{value}=\frac{\sum_{i=1}^{N}I\left(fraction\_miRNA<fraction\_random\left(i\right)\right)}{N}$$

For KIRC, the *p*-value was significant. Furthermore, we calculated the fractions of genomic regions with SCNAs across all genomes, which were shown to be higher than the fractions of miRNAs with SCNAs in all cancer types except for in KIRC samples. These results indicate that miRNAs tend to be present in SCNA regions in KIRC, but not in other cancer types.

### 2.2. Identification of miRNAs Associated with SCNAs

**Figure 2:**
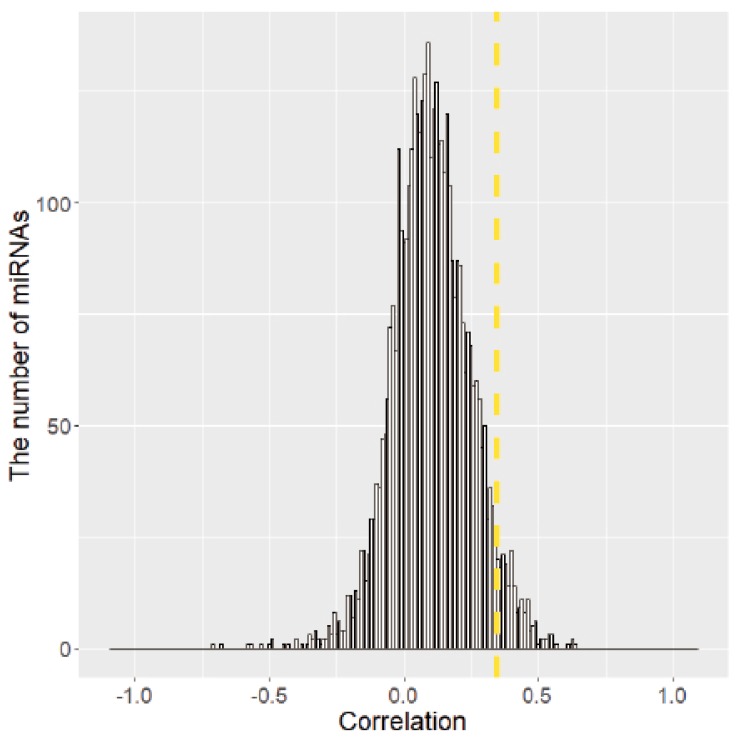
Relationship between copy numbers and miRNA expression. Pearson correlation coefficients (PCCs) showing the correlation between copy numbers and miRNA expression levels. Yellow dashed line, PCC threshold.

We investigated whether expression of any of the 971 miRNAs was associated with SCNAs. In Figure 2, the distribution of Pearson correlation coefficients (PCCs) of copy number values and expression of 971 miRNAs from all seven cancer types are presented. The PCCs for each cancer type are presented in Figure S1. Additionally, among the 971 miRNAs, we selected miRNAs in the SCNA region (copy number log2 ratio >0.25 or <−0.28) and examined the relationship between PCCs and miRNA SCNAs for each cancer type. Although these miRNAs were in SCNA regions, most showed weak correlations between expression levels and copy numbers. Scatter plots showing these correlations are presented in Figure S2.

**Figure 3:**
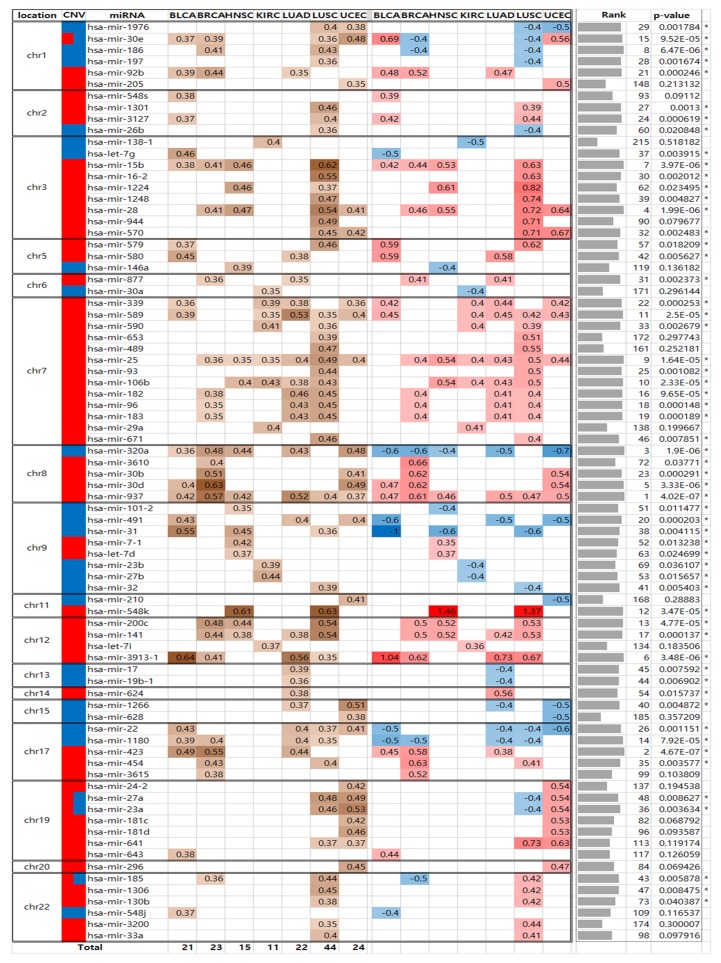
SCNA-miRNA heatmap obtained for seven cancer types. MiRNAs are listed according to their chromosomal locations. The PCC value, copy number state, and miRNA rank with *p*-value for each miRNA are shown (*, *p*-value < 0.05). The brown color intensities represent the degree of PCCs between miRNA copy numbers and expression levels, and red or blue intensities represent the average copy number values of samples amplified or deleted for the given miRNA and cancer type. The last row shows cancer-specific SCNA-miRNA numbers.

We identified somatic copy number alteration miRNAs (SCNA-miRNAs) by applying the conditions described in the Materials and Methods section. First, we drew a distribution of PCCs between the copy number values and miRNA expression levels using all miRNAs. The distribution is shown in Figure 2. The top α% = 5% value was used as a threshold, which corresponds to a PCC > 0.35. To determine the significance of the PCC value of 0.35, we additionally performed the following permutation test. For each miRNA, we calculated the pairwise PCCs between miRNA expression levels and randomly permuted copy number values. Next, we compared these PCCs to the observed PCCs. We repeated this process 1000 times for all miRNAs. The null hypothesis is that PCCs between miRNA expression levels and permuted copy numbers are the same as those between miRNA expression levels and original copy numbers. The *p*-value was 2.09 $\times 10^{-6}$, showing that the PCC value = 0.35 was significant (Figure S3). Next, as described in the Materials and Methods section, the thresholds for copy number amplification and deletion were selected as 0.25 and −0.28, respectively (β% = 10%; Figure 1A), and the fraction of samples with SCNAs >10% was used a threshold. As a result, we identified 80 SCNA-miRNAs and determined the regions in which they are localized (Figure 3). In BLCA, BRCA, HNSC, KIRC, LUAD, LUSC, and UCEC, 21, 23, 15, 11, 22, 44, and 24, respectively, SCNA-miRNAs were identified. We referred to these miRNAs as cancer-specific SCNA-miRNAs. Similar to the fractions of miRNAs in SCNA regions for each cancer type as shown in Figure 1C, the LUSC and KIRC samples contained the largest and smallest numbers of SCNA-miRNAs, respectively.

Figure 3 shows the list of 80 SCNA-miRNAs, their PCC values, and their copy number states. We examined the amplification and deletion states of these 80 miRNAs in seven cancer types; 26 and 15 miRNAs were amplified and deleted in a single cancer type, respectively, 27 and 8 miRNAs were consistently amplified and deleted across multiple cancer types, respectively, and four miRNAs had copy number states that differed depending on the cancer type (mir-30e, mir-27a, mir-23a, and mir-185).

### 2.3. Comparison between Pan-Cancer SCNA Regions and the SCNA-miRNA Regions

**Figure 4:**
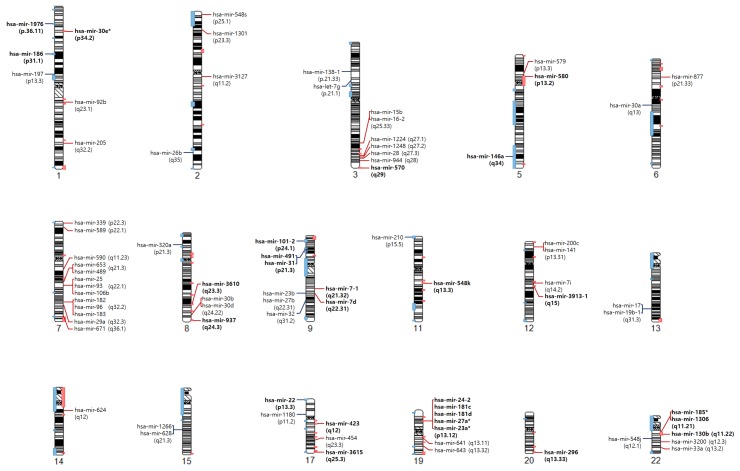
**Chromosome ideogram obtained by mapping SCNA-miRNAs and pan-cancer SCNA regions.** Pan-cancer SCNA regions, amplified (red) or deleted (blue), are shown for each chromosome, and the locations of the SCNA-miRNAs are indicated with cytogenetic bands. The 26 miRNAs in the pan-cancer SCNA regions are represented in bold, and the remaining 54 miRNAs are shown together, which are located within 10.6 mega bases (Mbs) of pan-cancer SCNA regions on average (ranging from 0.3 to 47.4 Mbs).

We compared genomic regions harboring SCNA-miRNAs with pan-cancer SCNA regions identified by GISTIC analysis [6]. Pan-cancer analysis revealed 140 recurrent focal SCNA regions from the copy number profiles of TCGA data sets that include 4934 primary cancer patients across 11 cancer types. These regions include known oncogenes and tumor suppressor genes. Figure 4 shows the pan-cancer SCNA regions and SCNA-miRNA regions. We observed 13 amplified regions including 18 miRNAs (mir-30e, mir-570, mir-580, mir-3610, mir-937, mir-548k, mir-3913-1, mir-423, mir-3615, mir-24-2, mir-181c, mir-181d, mir-27a, mir-23a, mir-296, mir-1306, mir-130b, and mir-185) and seven deleted regions including 8 miRNAs (mir-1976, mir-186, mir-146a, mir-101-2, mir-491, mir-31, mir-210, and mir-22).

When we investigated whether these specifically matched regions contain cancer-associated genes, 22 SCNA-miRNAs were found to be co-located with cancer-associated genes (Table 1). Cancer-associated genes were obtained from the Catalogue of Somatic Mutations in Cancer [31] database. Previous studies supported our results that most of the 22 SCNA-miRNA except for four miRNAs (hsa-mir-1976, hsa-mir-3913-1, hsa-mir-3615, and hsa-mir-1306) are related to cancer development [32,33,34,35,36,37,38,39]. However, few relationships were identified between miRNAs and co-located cancer-associated genes except for in the chr9p21.3 locus. The chr9p21.3 locus was shown to contain the tumor suppressor genes CDKN2A, mir-491, and mir-31. Li et al. [40] investigated the co-deletion of mir-491 and CDKN2A in glioblastoma and demonstrated that the deletion of mir-491 was not just a passenger event and that mir-491 acts as a tumor suppressor. Moreover, several studies showed that mir-31 and CDKN2A are concurrently deleted in cancers [41,42]. These results suggest that co-deletion of hsa-mir-491 and CDKN2A plays an important role in BLCA, LUAD, and UCEC, and that co-deletion of mir-31 and CDKN2A is a crucial event in BLCA and LUSC.

Figure 4 shows that most SCNA-miRNAs are located near pan-cancer SCNA regions. Furthermore, their copy number states (amplification or deletion) were similar to those in SCNA regions. Although many SCNA-miRNAs are co-located or proximal to cancer-related genes, their relationships have not been extensively investigated. Thus, it may be important to investigate whether miRNAs and co-located genes interact or if they independently affect oncogenesis.

### 2.4. Association between SCNA-miRNA and Survival of Cancer Patients

**Figure 5:**
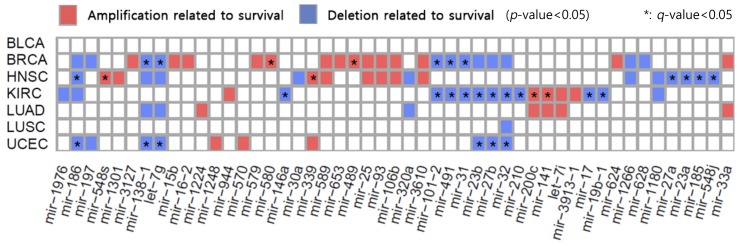
Survival analysis according to SCNA-miRNAs. Heatmaps showing miRNAs significantly affecting survival. Red and blue, the differences in patient survival when miRNA copy numbers were amplified and deleted, respectively.

The survival of cancer patients was analyzed to determine whether copy number changes of 80 SCNA-miRNAs were associated with death by cancer (Figure 5). Based on their copy numbers for each SCNA-miRNA, patients were divided into the amplification (or deletion) or unchanged group. The amplification (or deletion) group contained samples with a copy number value greater than 0.25 or lower than −0.28. Amplification and deletion thresholds were same as those described in Section 2.1. When the log-rank test was applied to the amplification and unchanged groups of patients, 25 SCNA-miRNAs were shown to significantly affect survival in at least one cancer type (*p*-value < 0.05). Similarly, 24 SCNA-miRNAs were shown to induce significant differences in survival between the deletion and unchanged groups (*p*-value < 0.05). After adjusting for multiple comparisons using the Benjamini-Hochberg procedure, 23 miRNAs exhibited significant differences with a *q*-value < 0.05. For the types of cancer associated with these SCNA-miRNAs, we present 32 pairs of SCNA-miRNAs and associated cancer types in relation to survival time based on the copy numbers of these miRNAs (Figure 5, Table S1). Notably, HNSC includes many subtypes arising from 12 tissue types. Therefore, we categorized 453 HNSC cancer patient samples based on tissue type and investigated the survival rates for four major tissue groups (oral tongue, oral cavity, larynx, and floor of the mouth).

Among the amplified miRNAs, two miRNAs (mir-141 and mir-200c) located close to each other in chromosomes were found to significantly affect the survival of patients with KIRC (*q* = 0.0032 for both miRNAs) and LUAD (*p* = 0.009 for both miRNAs). Associations between mir-141 expression and cancer development were previously reported [43,44], together with an association with the survival of patients with lung cancer [44]. Mir-200c was investigated as a potential prognostic factor in lung cancer [45].

For deleted miRNAs, for mir-31, the mean survival time of patients in the deletion group was shorter than that in the unchanged group of BRCA and KIRC patients (*q* = 0.0065 and 0.0024, respectively). A previous study suggested that downregulation of mir-31 and its host gene can be used as prognostic markers in breast cancer [46]. Additionally, the results of one study showed that expression of mir-31 was significantly reduced in gastric cancer tissues compared to that in non-tumor tissues [47]. Mir-491 copy number alterations were shown to affect the survival of patients with BRCA and KIRC (*q* = 0.0071 and 0.023, respectively). Hui et al. [48] showed that mir-491 is downregulated and acts as a tumor suppressor in ERα-positive breast cancer.

We investigated whether the 80 SCNA-miRNAs were more highly associated with the survival of cancer patients than the remaining 690 miRNAs (Supplementary Materials). Of the 560 pairs of SCNA-miRNAs and cancer types corresponding to the 80 SCNA-miRNAs, 5.4% were significantly associated with survival differences. Furthermore, of the 4,203 pairs of non-SCNA-miRNAs and cancer types corresponding to the 690 miRNAs, 2.8% were shown to significantly affect patient survival. This suggests that alterations in these SCNA-miRNAs with a *q*-value < 0.05 affect patient survival by more than other miRNAs (*p*-value = 0.00254, Fisher’s exact test). More specifically, HNSC and UCEC showed significant *p*-values (Table S2).

### 2.5. miR-589 as a New Potential Cancer Biomarker

When we examined SCNA-miRNAs related to survival and cancer types, we found that some miRNAs were not previously investigated. Among these, we specifically evaluated miR-589 in HNSC as an example of validation because its role in HNSC is unknown. In a previous study by Zhang et al., miR-589 was shown to be related to the aggressiveness of gastric cancer [49].

**Figure 6:**
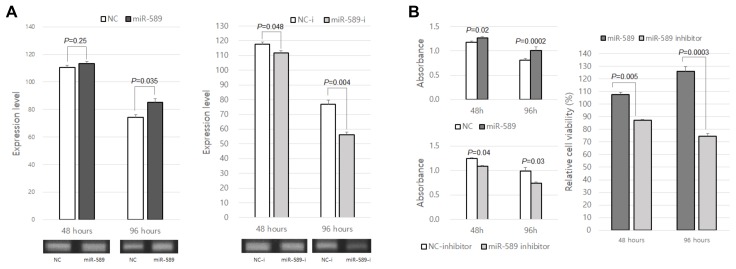
Effects of miR-589 on Hep-2 cells. The cells were transfected with miR-589, miR-589 inhibitor, and their negative controls. (**A**) Expression levels of miR-589 in the cells transfected with miR-589, miR-589 inhibitor, or their negative controls at 48 and 96 h. These are measured by RT-PCR, and 48 and 96 h were examined on different gels. Uncropped images of gels are shown in Figure S4. (**B**) Cell viability and proliferation rate. The transfected cells were measured by MTT assay at different time periods. The averages of three independent MTT assays performed in triplicate are shown with standard error (SE) bars. The *p*-values of miR-589 versus its normal control, miR-589 inhibitor versus its normal control, and miR-589 versus miR-589 inhibitor were calculated by using two-tailed *t*-test.

We experimentally validated the role of miR-589 in HNSC using the laryngeal cancer cell line Hep-2 cells (Figure 6, Figure S4). Details of the experiments are shown in the Supplementary Materials. The cells were transfected with miR-589, miR-589 inhibitor, and their negative controls for different time periods. Negative control cells of miR-589 were not transfected with miR-589, while negative control cells of miR-589 inhibitor were transfected with miR-589, but not with the miR-589 inhibitor. First, we examined miR-589 expression in these transfected cells by using reverse transcription (RT)-PCR. As presented in Figure 6A, miR-589 was shown to be more highly expressed in Hep-2 cells transfected with miR-589 than in the negative control, while the miR-589 expression was decreased in cells transfected with the miR-589 inhibitor compared to in the negative control of the inhibitor. Additionally, the cell viabilities and proliferation rates of these transfected cells were measured in a 3-(4,5-dimethylthiazol-2-yl)-2,5-diphenyltetrazolium bromide (MTT) assay (Figure 6B), and it was shown that relative cell viabilities of the cells transfected with miR-589 were significantly higher than those of cells transfected with the miR-589 inhibitor at both 48 and 96 h. The results of our in vitro experiment indicate that miR-589 plays a role in the proliferation of HNSC cells in vitro and that miR-589 is an HNSC oncogene.

### 2.6. Investigation of miRNAs Commonly Associated with SCNAs Across Seven Cancer Types

We examined whether the expression level of each miRNA was associated with SCNAs in several cancer types by using Fisher’s method [50]. Because we selected miRNAs with the percentage of samples used for PCC determination higher than 10% of total samples in each cancer type, we obtained PCCs for 595–630 miRNAs, depending on the cancer type, and determined the miRNA rankings for each cancer type. In total, 674 of 971 miRNAs were used to calculate the common rank value (CRV), which is the negative logarithm of the product of the uniformly distributed relative ranks. The results showed that 76 miRNAs were associated with SCNAs commonly across several cancer types (*p* < 0.05; Table S3). The top 10 miRNAs were mir-937, mir-423, mir-320a, mir-28, mir-30d, mir-3913-1, mir-15b, mir-186, mir-25, and mir-106b. By comparing the previously obtained 80 SCNA-miRNAs and these 76 miRNAs, 58 miRNAs were identified and found to be common to all cancers (CC-SCNA-miRNAs). These CC-SCNA-miRNAs and their rankings are presented in Figure 3 and are marked with asterisks.

### 2.7. CC-SCNA-miRNA Effects on mRNA and Protein Expression Levels

**Figure 7:**
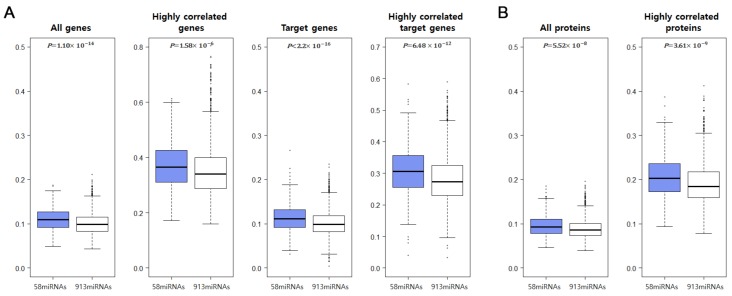
Boxplots showing the relationship between CC-SCNA-miRNAs and mRNA/protein expression. The mean absolute PCCs of CC-SCNA-miRNAs and genes were compared to the mean absolute PCCs obtained for the correlation between the other 913 miRNAs and gene expression. (**A**) Expression of all mRNAs, top 1% genes, target genes of miRNAs, top 5% target genes of miRNAs, and (**B**) all protein expression levels and top 20% proteins were considered.

We categorized 971 miRNAs into a group of 58 CC-SCNA-miRNAs and another group containing the remaining 913 miRNAs. For each group, we examined the effects of miRNAs on the expression levels of mRNAs, target genes of miRNAs, and proteins (Figure 7).

The results presented in Figure 7A demonstrate that higher absolute PCC values were obtained for CC-SCNA-miRNAs and mRNA expression levels than for 913 miRNAs when 20,530 human genes or target genes were considered (*p* < 0.05, *t*-test). Additionally, we investigated mRNAs with the top 1% of absolute PCCs and miRNA target genes with the top 5% of absolute PCCs obtained between miRNA and mRNA expression levels for each miRNA. The top 1% of mRNAs corresponded to 200 mRNAs, which is close to the average number of miRNA target genes in humans. Here, we observed that CC-SCNA-miRNAs were more highly correlated with these mRNAs than the other 913 miRNAs.

Furthermore, as shown in Figure 7B, the absolute PCCs obtained for miRNA and protein expression are presented. Depending on the cancer type, between 245 and 281 proteins were considered. The absolute PCCs showing a correlation between 58 CC-SCNA-miRNA and protein expression levels were significantly higher than those obtained for the other miRNAs (*p* < 0.05, *t*-test). Additionally, we chose the top 20% of absolute PCCs between the expression miRNAs and proteins and calculated their mean values for each miRNA. Here, absolute PCCs between CC-SCNA-miRNA and protein expression were significantly higher than those obtained in the other group. Taken together, this suggests that CC-SCNA-miRNAs affect the gene expression levels more than the other miRNAs.

To evaluate whether the high PCC values between miRNA and mRNA expression levels in CC-SCNA-miRNAs depends on the miRNA expression levels, we calculated the PCCs between miRNA and mRNA expression levels according to the miRNA expression levels. As a result, PCCs between mRNA and miRNAs were not related to the expression levels of SCNA-miRNAs or the remaining 913 miRNAs (Figure S5).

## 3. Discussion and Conclusions

We aimed to examine whether miRNAs tend to localize to SCNA regions in seven cancer types. As described in the Introduction section, previous studies addressing this issue showed different results [17,19,20]. These studies are described in detail in the Supplementary Materials. When we used a large-scale data set obtained from TCGA, our results demonstrated that miRNAs in patients with cancer are not more common in SCNA regions than in non-SCNA regions, except for in patients with KIRC. However, because SCNAs are very common genetic events, our study also showed that approximately 32−84% of the 971 miRNAs were in SCNA regions, depending on cancer types.

Because a large percentage of miRNAs are in SCNA regions, we focused on potential miRNAs, the expression of which is affected by SCNAs. Although the expression levels of many miRNAs were not correlated with their copy numbers, we identified 80 SCNA-miRNAs with expression levels that were significantly correlated with SCNAs. In Section 2.3, we compared regions containing SCNA-miRNA with pan-cancer SCNA regions. Twenty-six SCNA-miRNAs were found in the pan-cancer SCNA areas, representing 32.5% (26/80) of the SCNA-miRNAs. Additionally, we investigated non-SCNA-miRNAs in the pan-cancer SCNA regions, the copy numbers of which may not be correlated with their expression levels. Among the 891 non-SCNA-miRNAs, 135 (15.2%) were found in SCNA regions, indicating that the fraction of SCNA-miRNAs was greater than the fraction of non-SCNA-miRNAs in pan-cancer SCNA regions.

Our results demonstrate that SCNA-miRNAs are more closely associated with cancer survival compared to other miRNAs. To identify prognostic factors associated with cancer, univariate and multivariate analysis were performed (see details in the Supplementary Materials). The results of univariate analysis indicated that the pathological stages of five cancers (except LUSC and UCEC) were significant indicators of cancer (Table S4). However, most other variables were not clearly associated with cancer prognosis. For survival-significant SCNA-miRNAs with *q*-values < 0.05, 24 pairs were found to be significant by univariate analysis. In multivariate analysis of SCNA-miRNAs and clinical factors, all pairs, except for miR-548s and miR-185 and miRNAs in UCEC, were significant. This demonstrates that these SCNA-miRNAs are potential prognostic factors in cancer (Table S5).

Additionally, we ranked miRNAs associated with SCNAs across seven cancer types, and a literature search revealed that most of these miRNAs are associated with cancer development. To determine whether the SCNA-miRNAs are important in cancer development, we obtained cancer-related miRNA data from the Human MicroRNA Disease Database (HMDD) [39]. As a result, 51 SCNA-miRNAs and approximately 50% of the cancer-specific SCNA-miRNAs were found to be included in this database (Table S6). Furthermore, to identify additional BRCA-related miRNAs, we compared 23 BRCA-specific SCNA-miRNAs to BRCA-related miRNAs identified by Jin and Lee (2016) [51], who integrated three cancer-related features and prioritized miRNA ranking in BRCA samples. The result showed that nine miRNAs were included in the top 50 miRNAs, four miRNAs were ranked between 51 and 100, and the remaining 10 miRNAs were ranked in the top 500.

Moreover, we searched the SCNA-miRNAs shown to be cancer-related in the PubMed database. Among the 51 SCNA-miRNAs included in HMDD, we searched for literature stating that miRNAs that have high CRVs, indicating a significant correlation between SCNAs and miRNA expression levels in seven cancer types (see details in Supplementary Materials and Table S7). We also manually searched for studies related to the remaining 29 miRNAs, which were not included in the HMDD, in PubMed (Supplementary Materials and Table S7). Except for six SCNA-miRNAs (mir-548s, mir-653, mir-3610, mir-3913-1, mir-3615, and mir-1306), we confirmed that the other 22 miRNAs were cancer-related. Although a literature search did not yield any articles describing these six SCNA-miRNAs, they may be associated with cancer. Our results indicated that the expression levels of miR-3913-1, miR-3615, and miR-1306 were positively correlated with the expression of co-located cancer-related genes, while that of miR-3610 was not correlated with any co-located cancer-related gene (Table 1). Thus, further studies are needed to determine whether co-location of these miRNAs and cancer-related genes is a random occurrence or if the miRNAs play an active role in cancer development.

We further showed that the SCNA-miRNAs target a larger number of genes than other miRNAs and the expression levels of genes targeted by SCNA-miRNAs were correlated more with the expression levels of these miRNAs, confirming that SCNA-miRNAs play important roles in oncogenesis.

Here, we used different numbers of samples for each cancer type, and the numbers of tumor and normal samples were not equal. Although for cancer samples, each cancer type included at least 150 samples, the number of normal samples used to obtain mRNA expression data was relatively small for some cancer types such as UCEC. Using a smaller number of normal samples may have affected the normalization of expression levels of genes and the identification of differentially expressed genes. However, genes showing significant differences between tumor samples and normal samples may have been incorporated in our analysis.

In conclusion, our results revealed that SCNA-miRNAs may significantly affect biological process related to cancer development, confirming the importance of SCNAs in non-coding regions in cancer development and progression.

## 4. Materials and Methods

### 4.1. Datasets

We downloaded DNA copy number, miRNA expression, mRNA expression, and protein expression data sets of cancer samples from TCGA data portal [21,22,23,24,25,26,27]. MiRNA, mRNA, and protein expression data were generated by IlluminaHiSeq_miRNASeq, IlluminaHiSeq_RNASeqV2 (San Diego, CA, USA), and M.D. Anderson Reverse Phase Protein Array 61 Core, respectively. The miRNA and mRNA expression data of unmatched normal samples were obtained from Hamilton et al. [4], which contain the normal samples for the seven cancer types. Tumor sample numbers for DNA copy number, miRNA expression, mRNA expression, and protein expression analyses of BLCA, BRCA, HNSC, KIRC, LUAD, LUSC, and UCEC were 247, 702, 453, 235, 418, 327, and 156, respectively. Normal sample numbers for miRNA expression data in BLCA, BRCA, HNSC, KIRC, LUAD, LUSC, and UCEC were 16, 83, 38, 70, 45, 35, and 28, respectively, and those for mRNA expression data were 13, 104, 37, 67, 57, 17, and 5, respectively. For DNA copy numbers, we used level 3 segmented copy number data obtained from TCGA generated using the Affymetrix Genome-Wide Human SNP Array 6.0 (Santa Clara, CA, USA).

### 4.2. Data Pre-Processing

We obtained a list of 1,046 miRNAs from TCGA data sets, where the genomic locations of miRNA precursors were obtained from the miRBase [28] when their expression values were measured by IlluminaHiSeq_miRNASeq. Thus, to obtain the DNA copy numbers of miRNAs, we determine the genomic location of miRNA precursors using the miRBase [28] (v21, June 2014), which was annotated using the hg19 human reference genome. Next, of 1,046 miRNAs with genomic locations, we used 971 miRNAs with their copy numbers and expression values throughout this study.

MiRNA and mRNA expression values were normalized using the normal samples as follows:

(1) $$y_{ij}=\log{}_{2}\frac{cancer{}_{ij}}{{normal}_{i}}$$

where $cancer{}_{ij}$ represents the expression of miRNA/mRNA *i* in the *j*th cancer sample and ${normal}_{i}$ is the average value of the miRNA/mRNA *i* expression in normal samples.

### 4.3. Identifying SCNA-Associated miRNAs

To identify SCNA-associated miRNAs, we investigated whether the copy number of a region containing an miRNA was amplified or deleted in a certain fraction of samples, if the miRNA is differentially expressed, and if the copy number was highly correlated with miRNA expression. Thus, we obtained copy number values for genomic regions containing miRNAs. We considered the miRNAs satisfying the following three conditions in at least one cancer type as SCNA-miRNAs.

First, we investigated the copy numbers of miRNA gene locations in all cancer samples. We set the top and bottom β% values as significant copy number amplification and deletion thresholds. We then selected miRNAs in genomic regions with significant copy number amplifications or deletions in at least γ% of samples. Furthermore, we calculated PCCs between the copy number values and miRNA expression levels. We selected miRNAs with the top α% PCC values. Here, we filtered out miRNAs for which the number of samples used to calculate the PCCs between SCNAs and miRNA expressions was less than 10% of the total samples. Third, we selected differentially expressed miRNAs with a *q*-value < 0.01 by comparing cancer samples with normal samples using a *t*-test and Bonferroni multiple comparison correction.

### 4.4. Identification of miRNAs Commonly Associated with SCNAs Across Seven Cancer Types

We investigated miRNAs commonly associated with SCNAs across multiple cancer types based on PCCs between the copy number values and miRNA expression levels. We filtered out miRNAs for which the number of samples used to calculate PCCs between SCNAs and miRNA expression was lower than 10% of the total samples of each cancer. To control bias from miRNAs that are significantly correlated with SCNAs in certain cancer types, we first determined miRNA ranks based on the PCCs for each cancer type using the following formula:

(2) $${rr}_{\mu ,k}=\frac{r_{\mu ,k}}{|N_{k}|}$$

where $r_{\mu ,k}$ is a rank of PCC for miRNA μ and cancer type *k*, and $N_{k}$ is the number of miRNAs with PCC values in the given cancer type *k*. To evaluate whether an miRNA μ was generally associated with SCNAs across seven cancer types, we calculated CRVs for each miRNA:

(3) $${\mathrm{CRV}}_{\mu }=-2\sum_{k}ln\left(rr_{\mu ,k}\right)$$

Under the null hypothesis in which there is no association between copy number and expression of miRNA across all cancer types, we assumed that the CRV followed a $\chi^{2}$ distribution with 14 (2 × cancer type numbers) degree of freedom, which follows from Fisher’s method [50,52]. As a result, we identified miRNAs affected by SCNAs across seven cancer types with *p*-values < 0.05.

### 4.5. miRNA Effects on mRNAs and Proteins

To investigate the influence of miRNAs on mRNA and protein expression, we used miRNA, mRNA, and protein expression datasets. We calculated PCCs between miRNA and mRNA expression levels and PCCs between miRNA and protein expression levels in all tumor samples.

For each miRNA, PCCs between the miRNA and mRNA expression levels of 20,530 genes were calculated and the mean absolute value of PCCs was determined. Using the same method, we analyzed the relationships between miRNA-miRNA target genes and miRNA-proteins. Experimentally validated miRNA-target gene information was obtained from miRTarBase [53]. TCGA protein expression data were obtained from the GDC Data Portal (https://portal.gdc.cancer.gov/), which were generated by M.D. Anderson Reverse Phase Protein Array 61 Core. The numbers of proteins were 245, 281, 274, 273, 276, 276, and 245 in BLCA, BRCA, HNSC, KIRC, LUAD, LUSC, and UCEC, respectively.

### 4.6. Survival Analysis

In this study, we investigated the survival of patients according to copy number alterations. Clinical data for all samples were obtained from TCGA, and we examined the status of patients (‘dead’ or ‘alive’) and last follow-up. Cancer patients were divided into two groups based on their copy number levels in each miRNA region: amplification group (or deletion group) and unchanged group. The amplification and deletion groups contained samples with copy number values greater than the copy number amplification threshold and lower than the copy number deletion threshold, respectively. For samples in the unchanged group, copy number values were between the amplification and deletion thresholds. Next, using the log-rank test, we obtained *p*-values showing significant differences in time until death between the two groups of patients with cancer for each miRNA. To correct for multiple comparisons, the Benjamini-Hochberg procedure was used to calculate the false discovery rate as *q*-values.

## Figures and Tables

**Table 1:** SCNA-miRNA regions and exactly matched pan-cancer SCNA regions with cancer-related genes. (The degree of correlation between miRNAs and cancer-related genes is marked with asterisks: * (0.1–0.2), ** (0.2–0.3), *** (>0.3) in at least one cancer type).

Chr.	Genomic Location	Cytoband	miRNA	State	Cancer-Related Genes
chr1	26554542-26554593	1p36.11	hsa-mir-1976	Deleted	ARID1A **, SDHB ***
	40754355-40754446	1p34.2	hsa-mir-30e	Amplified	MYCL ***
	71067631-71067716	1p31.1	hsa-mir-186	Deleted	
chr3	195699401-195699497	3q29	hsa-mir-570	Amplified	TFRC ***
chr5	36147892-36147988	5p13.2	hsa-mir-580	Amplified	IL7R, LIFR *
	160485352-160485450	5q34	hsa-mir-146a	Deleted	EBF1 **, FGFR4 **, FLT4 *, ITK ***, NPM1 *, TLX3, NSD1, RANBP17 **, PWWP2A ***
chr8	116874728-116874800	8q23.3	hsa-mir-3610	Amplified	
	143812957-143813042	8q24.3	hsa-mir-937	Amplified	NDRG1 ***, RECQL4 ***
chr9	4850297-4850375	9p24.1	hsa-mir-101-2	Deleted	NFIB ***
	20716105-20716188	9p21.3	hsa-mir-491	Deleted	CDKN2A**
	21512115-21512185		hsa-mir-31	Deleted	
chr11	568089-568198	11p15.5	hsa-mir-210	Deleted	HRAS *, CARS *
	70283955-70284070	11q13.3	hsa-mir-548k	Amplified	*CCND1*
chr12	69584722-69584823	12q15	hsa-mir-3913-1	Amplified	MDM2 ***, HMGA2 **, PTRRB
chr17	1713903-1713987	17p13.3	hsa-mir-22	Deleted	YWHAE ***
	30117079-30117172	17q12	hsa-mir-423	Amplified	ERBB2 *, CDL12, NF1 **, RARA
	74748613-74748699	17q25.3	hsa-mir-3615	Amplified	RNF213*
chr19	13836287-13836359	19p13.12	hsa-mir-24-2	Amplified	BRD4 ***, LYL1 ***
	13836440-13836517		hsa-mir-27a	Amplified	
	13836587-13836659		hsa-mir-23a	Amplified	
	13874699-13874808		hsa-mir-181c	Amplified	
	13874875-13875011		hsa-mir-181d	Amplified	
chr20	58817615-58817694	20q13.33	hsa-mir-296	Amplified	
chr22	20033139-20033220	22q11.21	hsa-mir-185	Amplified	BCR ***, SEPT5 **, MAPK1, LZTR1 ***, CLTCL1 ***
	20086058-20086142		hsa-mir-1306	Amplified	
	21653304-21653385	22q11.22	hsa-mir-130b	Amplified	

## Supplementary Materials

The following are available online at http://www.mdpi.com/2072-6694/10/12/475/s1, Figure S1: The distribution of PCCs showing the correlation between the copy numbers and expression of 971 miRNAs in BLCA, BRCA, HNSC, KIRC, LUAD, LUSC, and UCEC samples. Figure S2: Scatter plots showing PCCs between miRNA copy numbers and expression levels across miRNA copy numbers for each cancer type. Figure S3: Distribution of PCCs showing a correlation between randomly permuted copy number values and miRNA expressions. Figure S4: Uncropped gel images for data in Figure 6. Figure S5: Scatter plots showing PCC between miRNA and mRNA expression levels according to miRNA expression. Table S1: Analysis of patient survival according to copy numbers of the 84 pairs of SCNA-miRNAs and cancer types. Table S2: Comparison of survival times between the groups with SCNA-miRNAs and other miRNAs according to miRNA copy numbers. Table S3: miRNAs commonly correlated with SCNAs across seven cancer types. Table S4: Univariate analysis of clinical factors and miRNAs to examine prognostic factors associated with cancer. Variables differ depending on cancer types. Table S5: Multivariate analysis of clinical factors and miRNAs to examine prognostic factors associated with cancer. Table S6: Cancer-related miRNAs according to the HMDD. Table S7: SCNA-miRNAs reported in HMDD and the results of literature search demonstrating SCNA-miRNA associations with cancer development and progression.

## Abbreviations

The following abbreviations are used in this manuscript:

BLCA	Urothelial bladder carcinoma
BRCA	Breast invasive carcinoma
CC-SCNA-miRNAs	SCNA-miRNAs common for all cancers
SCNA-miRNAs	Copy number variable miRNAs
CRV	Common rank value
FRA	Fragile sites
HNSC	Head and neck squamous cell carcinoma
KIRC	Kidney renal clear cell carcinoma
LUAD	Lung adenocarcinoma
LUSC	Lung squamous cell carcinoma
PCCs	Pearson correlation coeffients
SCNAs	Somatic copy number alterations
TCGA	The cancer genome atlas
UCEC	Uterine corpus endometrial carcinoma
