# Supplementary material for: Identification and Characterization of MicroRNAs Associated with Somatic Copy Number Alterations in Cancer
Source: Cancers (Basel). 2018 Nov 29;10(12):475. doi: 10.3390/cancers10120475 (PMC6315597; doi:10.3390/cancers10120475)
Supplement: Supplementary file 1 [file cancers-10-00475-s001.pdf]

cancers

MDPI

Supplementary Materials

Identification and Characterization of MicroRNAs Associated with Somatic Copy Number Alterations in Cancer

Jihee Soh, Hyejin Cho, Chan-Hun Choi and Hyunju Lee

Contents

1. Comparison of previous studies -- 3

2. Univariate and multivariate analysis -- 3

3. PubMed literature search of cancer-related SCNA-miRNAs ------------------- 3

4. Description of materials and methods used in survival analysis ------------- 4

4.1. miRNA information --- 4

4.2 Cell proliferation and miRNA expression analysis ------------------------------------ 5

5. Supplementary Figures -- 6

5.1. Figure S1 -- 6

5.2. Figure S2 -- 7

5.3. Figure S3 -- 8

5.4. Figure S4 -- 9

5.5. Figure S5 -- 11

6. Supplementary Tables -- 12

6.1. Table S1 -- 12

6.2. Table S2 -- 16

6.3. Table S3 -- 18

6.4. Table S4 -- 20

6.5. Table S5 -- 22

6.6. Table S6 -- 28

6.7. Table S7 -- 29

7. References -- 32

1. Comparison of previous studies

A previous analysis by Calin et al.[1)] investigating 186 miRNAs based on 296 PubMed articles showed that miRNAs tend to localize to FRAs. Zhang et al.[2)] investigated SCNA regions for 283 known human miRNA genes in breast cancer, ovarian cancer, and melanoma, and they considered miRNAs observed in >15% of tumor samples with SCNAs as those located in SCNA regions. The result showed that 37.1%, 72.8%, and 85.9% of miRNAs were located in SCNA regions in ovarian cancers, breast cancers, and melanomas, respectively. Especially, in ovarian cancer, out of 78 miRNAs involved in genomic alterations, expression levels of 57 (73.1%) miRNAs were concordantly related with DNA copy numbers. In contrast to the results of these studies[1,2)], when Lamy et al.[3)] investigated relationship between copy numbers and expression levels of 18 and 41 differentially expressed miRNAs in colon and prostate cancers, respectively, they did not find any statistically significant relationship. The details of these studies are summarized in the following table.

References	# of cancer types	# of samples or articles	Copy number thresholds	# of investigated miRNAs	# of amplified and deleted miRNAs
Calin et al. [1)]	Variety of cancer types from PubMed	296 PubMed articles	154 minimally deleted and amplified regions (median length 4.14 and 2.45 Mb, respectively) were found from 296 PubMed papers.	186 miRNAs	80 miRNAs
Zhang et al. [2)]	3 (ovarian, breast cancer, and melanoma)	227 samples	The fluorescence intensity ratio of tumor to reference DNA < 0.8 was considered copy number loss, and > 1.2 was a gain.	283 miRNAs	41 miRNA with gene copy number changes (26 with gains and 15 with losses)
Lamy et al. [3)]	3 (prostate, bladder, and colon cancers)	271 samples	A threshold of ±2 s.d. (above 2 s.d.: gain, below 2 s.d.: loss)	283 miRNAs	106, 104 and 53 miRNAs for prostate, colon, and bladder cancers, respectively.

2. Univariate and multivariate analysis

Multivariate analyses including SCNA-miRNAs and clinical factors were performed using a Cox proportional hazard model, in order to identify prognostic factors associated with cancer. Before multivariate analyses, univariate analyses for various clinical factors, including gender, history of other malignancies, clinical tumor, node, and metastasis stages, pathological stages, age at initial pathological diagnosis (< 60 or ≥ 60), the number of pack-years for smokers (BLCA, LUAD, LUSC, and HNSC), alcohol history (HNSC), and estrogen and progesterone receptor status (BRCA), were evaluated as factors associated with cancer prognosis.

3. PubMed literature search of cancer-related SCNA-miRNAs

We searched to identify the SCNA-miRNAs shown to be cancer-related in the PubMed. Among the 51 SCNA-miRNAs included in HMDD, we searched for literature evidences for miRNAs that have high CRVs, indicating a significant correlation between SCNAs and miRNA expression levels in seven cancer types. We found that mir-320a (ranked third), mir-28 (fourth), mir-15b (seventh), and mir-25 (ninth), mir-106b (tenth), mir-30e (15th), mir-141 (17th), and mir-22 (26th) were reported as

important cancer-related miRNAs in at least three cancer types. Mir-320a and mir-22 were shown to have tumor suppressor functions in most cancer types. Wang et al.[4)] discovered that mir-320a suppresses proliferation, migration, and invasion of breast cancer cells by targeting RAB11A, while Ling et al.[5)] experimentally verified that mir-22 inhibits lung cancer growth, and showed the association between ErbB3 oncogene and mir-22. Many studies demonstrated the oncogenic roles of mir-28, mir-15b, mir25, mir-106b, and mir-141. Mir-28 was showed to be a HNSC and LUSC biomarker[6,7)]. Mir-15b was shown to be differentially expressed in many cancer types[8-11)]. Aherne et al.[12)] reported that mir-25 affects MEK and TRAIL expressions and directly or indirectly regulates the expression of almost 100 genes. Additionally, crucial roles of mir-25 in BRCA and lung cancers were identified by several studies[13,14)], while a miRNA profiling showed the dysregulation of mir-106b in several cancer types[15,16)]. Tejero et al.[17)] reported that the overexpression of mir-141 is associated with shorter overall survival in lung cancer. Yang et al.[18)] examined the functions of mir-28, shown to negatively regulate the expression of Nrf2 that suppresses oxidative stress in BRCA. Furthermore, the upregulation or downregulation of mir-30e was observed in different cancer types[19-22)].

We also manually searched for literature concerning the remaining 29 miRNAs, which were not included in HMDD, in PubMed. Except for six SCNA-miRNAs (mir-548s, mir-653, mir-3610, mir-3913-1, mir-3615, and mir-1306), we confirmed that other 22 miRNAs were described as cancer-related. Although mir-937 was ranked first among the CC-SCNA-miRNAs and showed high PCC value between SCNA and miRNA expressions in all cancer types but one, it was not included in HMDD and only one study reported that mir-937 promotes cell proliferation in lung cancer[23)]. Zhang et al.[24)] showed that mir-589 (ranked 11th) directly targets MAP3K8 and inhibits the stemless of hepatocellular carcinoma. Gross et al.[25)] showed the relationship between TP53-3p events and mir-548k (ranked 12th) expression in HNSC, while Ge et al.[26)] reported the effects of mir-1180 (ranked 14th) in BLCA. The role of mir-92b (ranked 21th) was examined in several studies[27-29)], and Sun et al.[30)] investigated the role of mir-3127 (ranked 24th) in lung cancer. Fang et al.[31)] elucidated the role of mir-1301 (ranked 27th) that inhibits cell proliferation, migration, and invasion in HepG2 cells.

4. Description of materials and methods used in survival analysis

4.1. miRNA information

Although the TCGA miRNA expression data include 1046 miRNAs, some miRNAs do not have expression values. Thus, we selected 971 miRNAs having both miRNA expression values and copy number values. However, expression values of some miRNAs are zero for all cancer or normal samples in particular cancer types. Thus, in the survival analysis, we filtered out the miRNAs having zero expression values when selecting non-SCNA-miRNAs, resulting in different numbers of miRNAs depending on cancer types, as shown in the following table. As a result, a total of 770 miRNAs and 690 (= 770 – 80) non-SCNA-miRNAs were used for the survival analysis. Thus, 4203 pairs of non-SCNA-miRNAs and cancer types were obtained as shown in the following table.

	# of miRNAs after filtering miRNAs whose expression values are zero for all cancer or normal samples	SCNA-miRNA	Non-SCNA-miRNA
**BLCA**	630	80	550
**BRCA**	697	80	617
**HNSC**	708	80	628
**KIRC**	654	80	574
**LUAD**	685	80	605

**LUSC**	699	80	619
**UCEC**	690	80	610
**Union of seven cancers**	770	80	690
**# of pairs of miRNA and cancer type cancers**		560	4203

4.2 Cell proliferation and miRNA expression analysis

The human laryngeal cancer cell line Hep-2 was obtained from Korean Cell Line Bank (Seoul, South Korea), and cultured in Dulbecco's Modified Eagle's Medium (DMEM; Lonza, USA) supplemented with 10% fetal calf serum (FCS; Gibco, USA) and 1% antibiotic/antimycotic reagent (Sigma-Aldrich, USA). The cells were maintained in a humidified incubator with 5% CO_2 at 37℃ for growth.

Hsa-miR-589-5p mimics and hsa-miR-589-5p inhibitors were purchased from Genolution (Seoul, South Korea). For transfection, cultured cells were removed after the trypsin-EDTA (Sigma-Aldrich, USA) treatment, and centrifuged (Vision Scientific, South Korea) in phosphate-buffered saline (PBS; Gibco, USA). Afterwards, these cells were seeded into 24-well plates (4×10^4 cells/well), and transfected with the miRNAs using G-fectin, a RNAi transfection reagent (Genolution, South Korea). The transfected cells were cultured in regular culture medium for 48 and 96 h before further analyses.

MiRNA expression in the transfected cells at 48 and 96 h was verified using reverse transcription polymerase chain reaction (RT-PCR). Total RNA was isolated using TRIzol reagent (Gibco-BRL, USA). The isolated RNAs dissolved in diethylpyrocarbonate (DEPC)-treated water (Biosolution, Korea), and were quantified using Biophotometer (Eppendorf, Germany) at 260 nm. Reverse transcription of the total RNA was primed using MiRNA First-Strand Synthesis kit (Takara, USA). The designed PCR primers used in this study were as follows: hsa-mir-589-5p forward primer, 5′-ACCCTGGTCTGCACTCTATC-3′; hsa-mir-589-5p reverse primer, the universal mRQ 3′ primer (Takara, USA); forward primer of the small nuclear RNA (snRNA) U6 used for a housekeeping gene, 5′-GGGCAGGAAGAGGGCCTAT-3′; and reverse primer of snRNA U6, 5′-AAAAATATGGAACGCTTCACGAATTTG-3′. MiRNA expression levels were determined using Gel Imaging System (Davinch-K, South Korea).

The cell proliferation rates of the transfected cells were evaluated by the 3-(4,5-dimethylthiazol-2-yl)-2,5-diphenyltetrazolium bromide (MTT) assay. We added 50μL of MTT reagent (5 mg/mL; Amresco, USA) to each well, and incubated at 37℃ for 4 h. Afterwards, the medium was removed, and 400μL of DMSO (Amresco, USA) were added to each well. After shaking the plates for 10 min, the optical densities were measured at 570 nm using microplate reader (Bio-Rad, USA).

5. Supplementary Figures

5.1. Figure S1

The distribution of PCCs showing the correlation between the copy numbers and expression of 971 miRNAs in BLCA, BRCA, HNSC, KIRC, LUAD, LUSC, and UCEC samples.

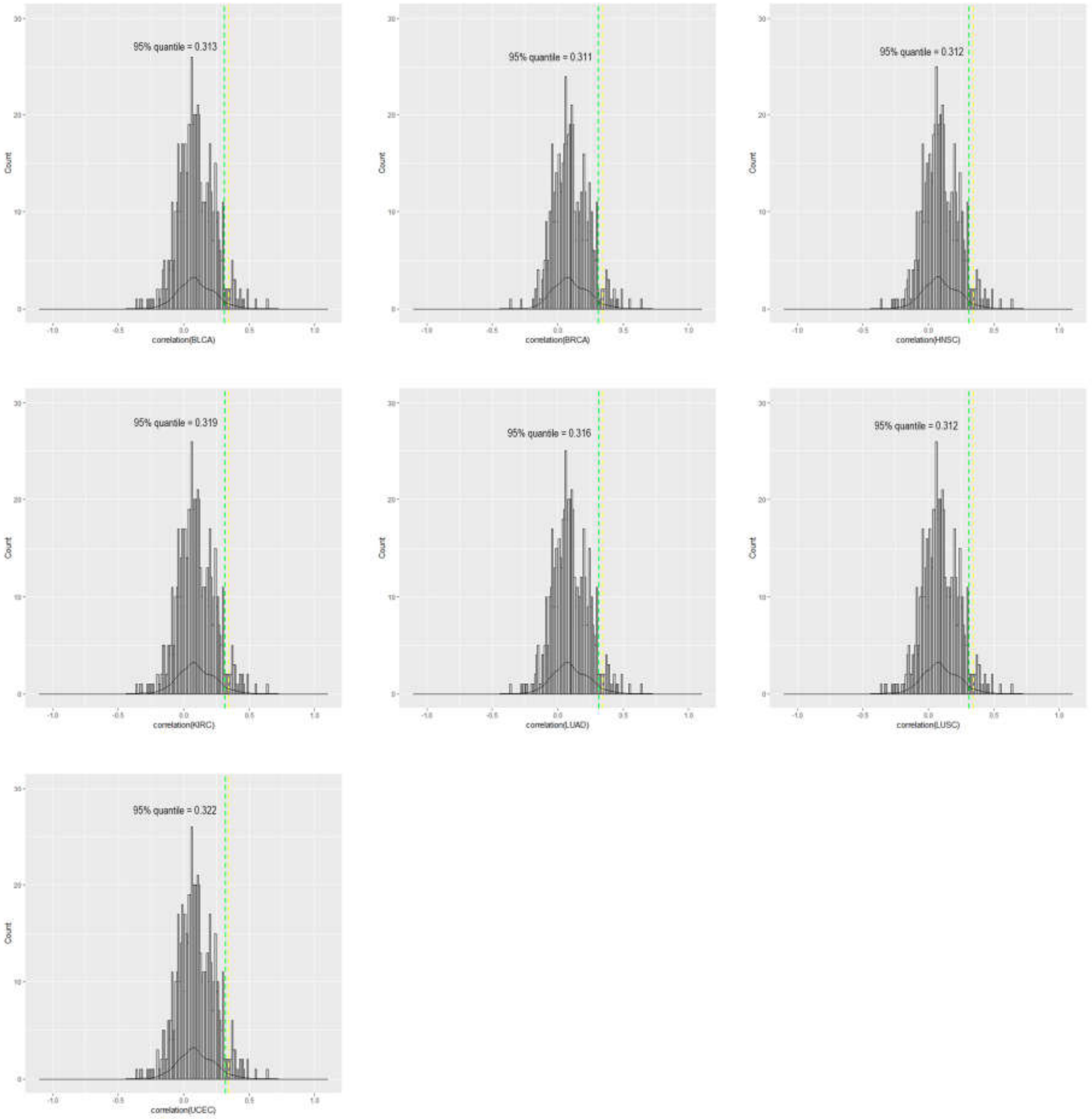

5.2. Figure S2.

Scatter plots showing the relationship between PCCs and miRNA copy numbers for each cancer type. Each dot indicates a miRNA, while the vertical dashed line shows the PCC threshold. Yellow, red, and blue lines represent the copy number mean, the amplification threshold, and the deletion threshold, respectively.

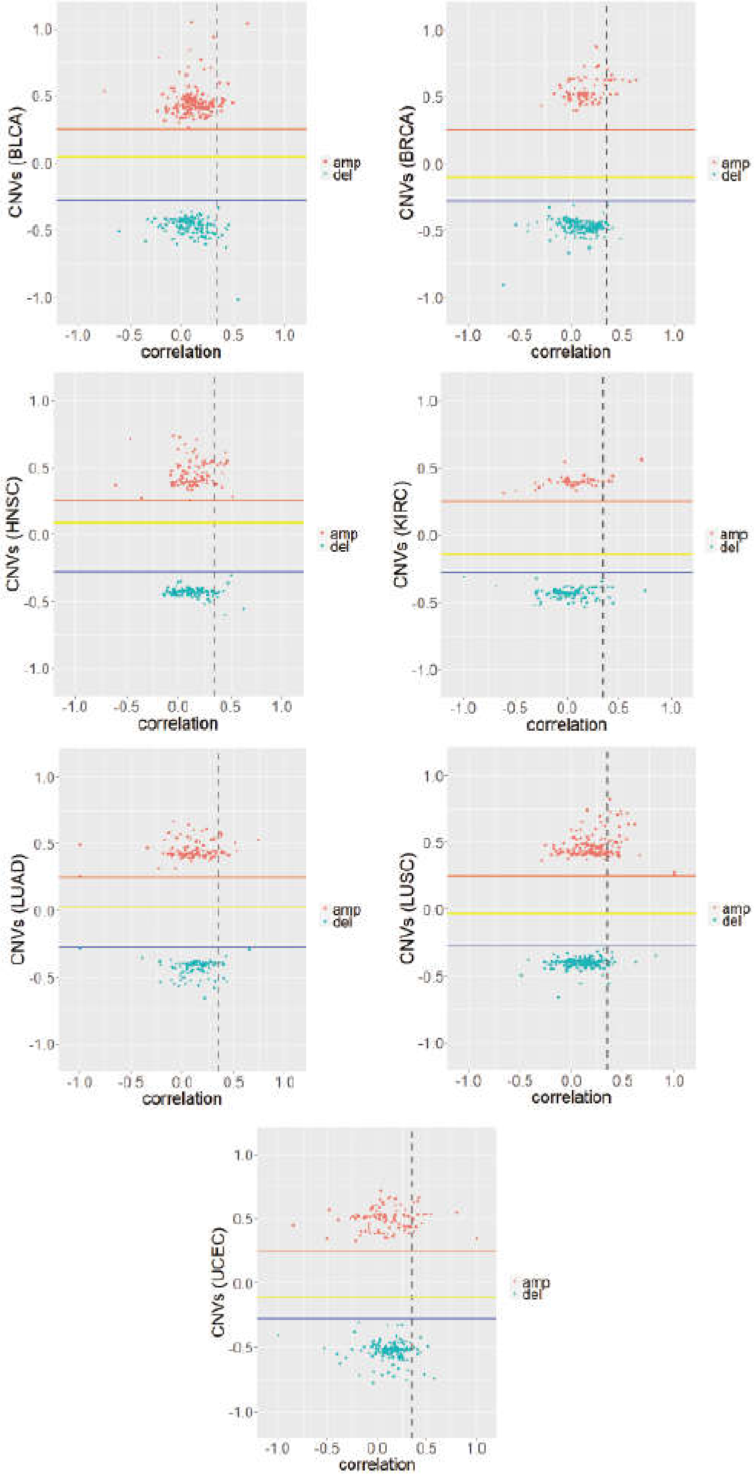

5.3. Figure S3.

Distribution of PCCs showing a correlation between randomly permuted copy number values and miRNA expressions.

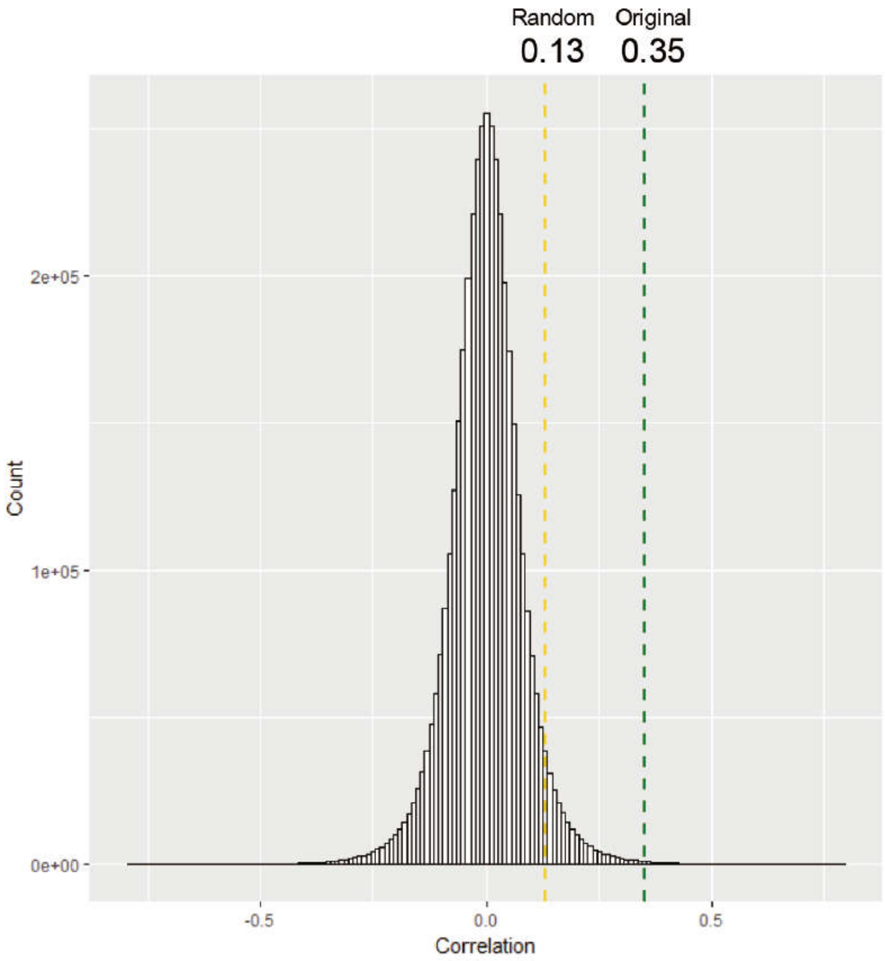

5.4. Figure S4.

Uncropped gel images for data in Fig. 6. (a) The expression levels of hsa-miR-589-5p, hsa-miR-589-5p inhibitor, and their negative controls in Hep-2 cells are shown for 48h and 96h. For 48h and 96h, these were measured on different gel. (b) The expression levels of U6 in Hep-2 cells transfected with hsa-miR-589-5p, hsa-miR-589-5p inhibitor, and their negative controls were measured, which was processed on the same gel for 48h and 96h.

(a)

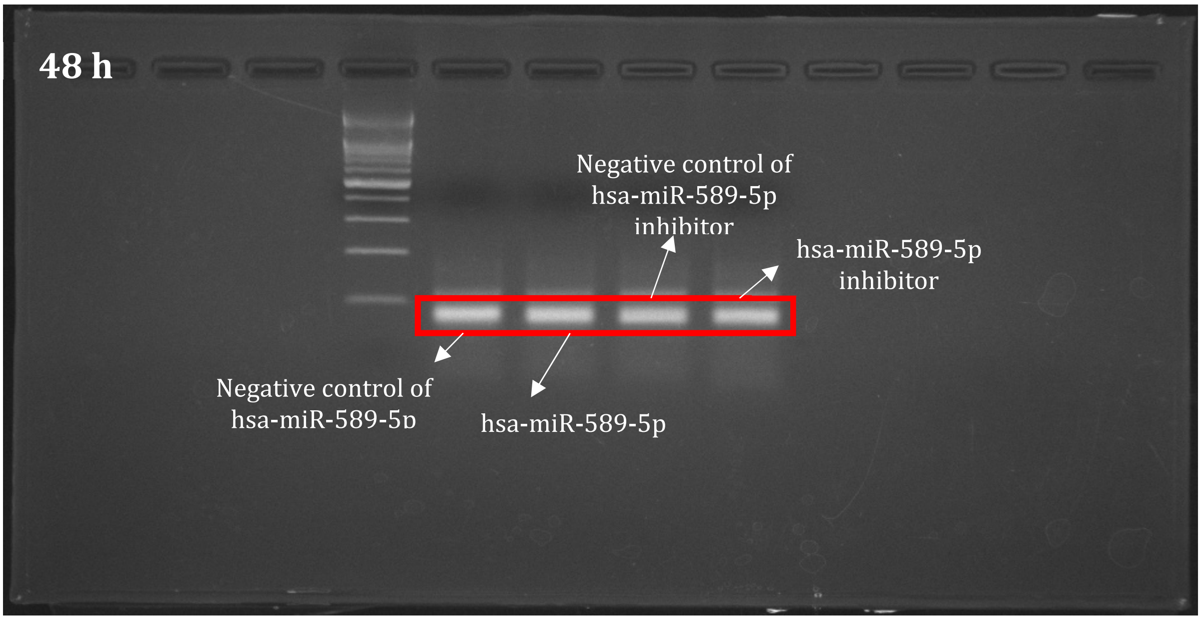

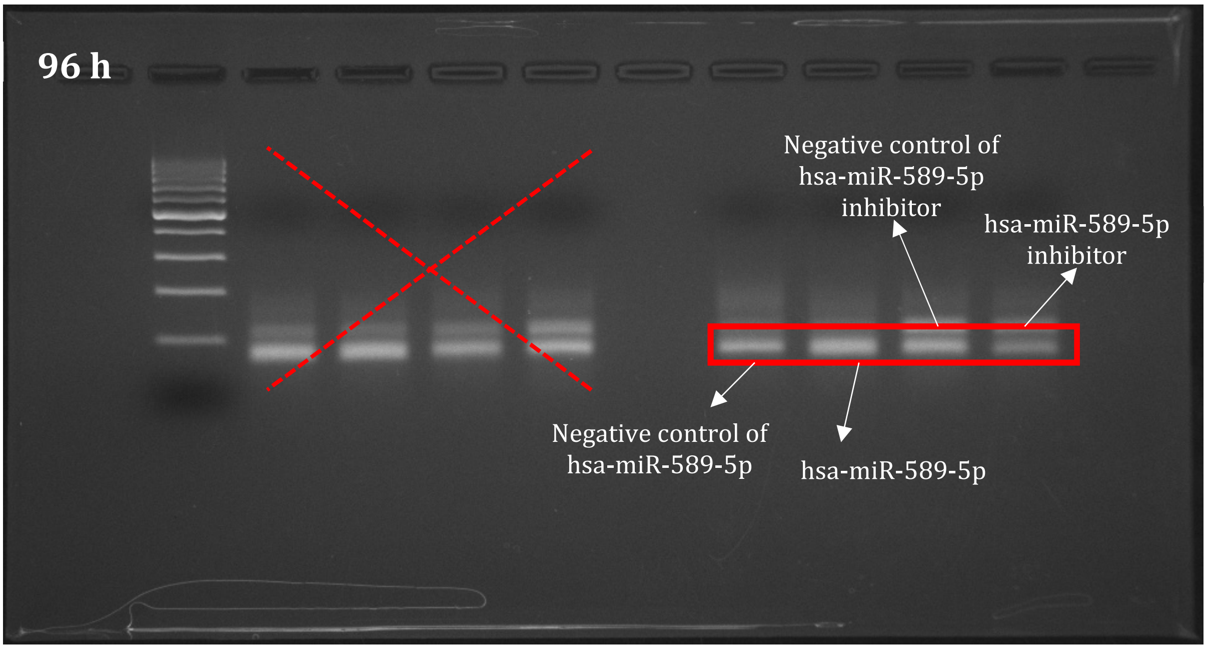

(b)

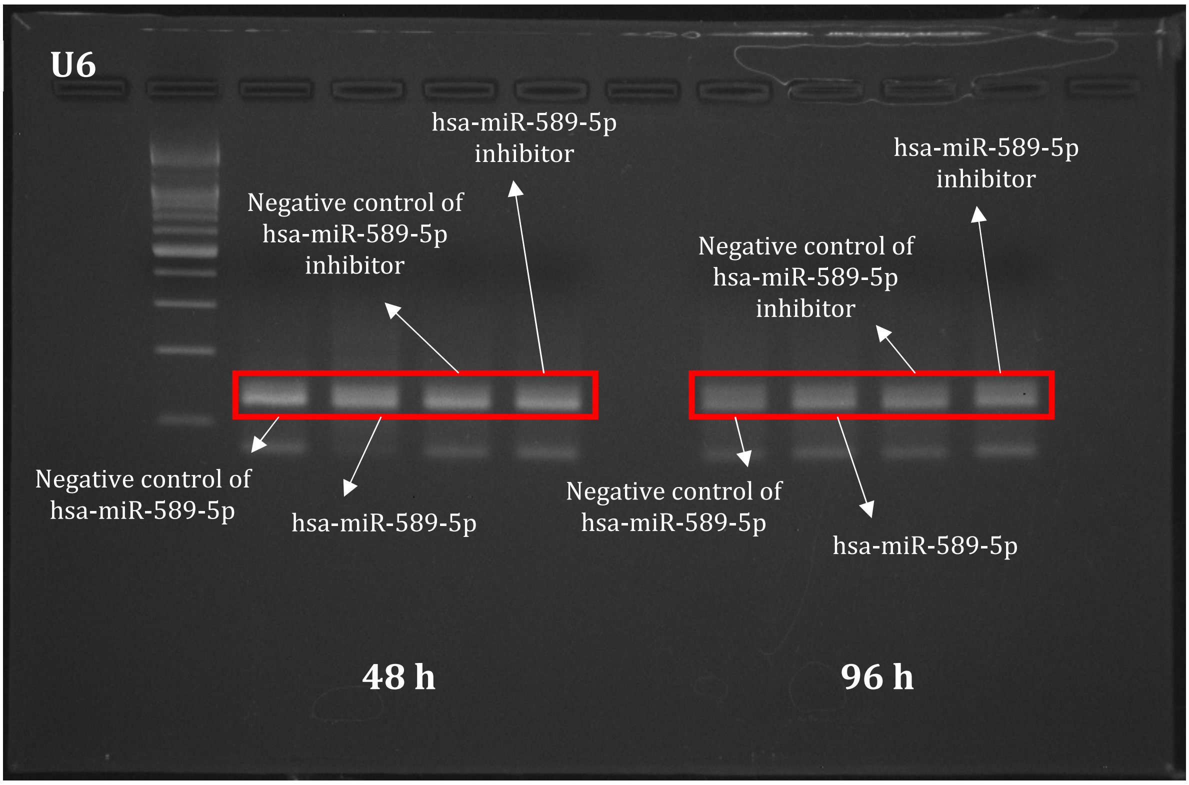
U6
hsa-miR-589-5p inhibitor
Negative control of hsa-miR-589-5p inhibitor
Negative control of hsa-miR-589-5p
hsa-miR-589-5p
hsa-miR-589-5p inhibitor
Negative control of hsa-miR-589-5p inhibitor
Negative control of hsa-miR-589-5p
hsa-miR-589-5p
48 h
96 h

5.5. Figure S5.

Scatter plots showing PCC between miRNA and mRNA expression levels according to miRNA expression.

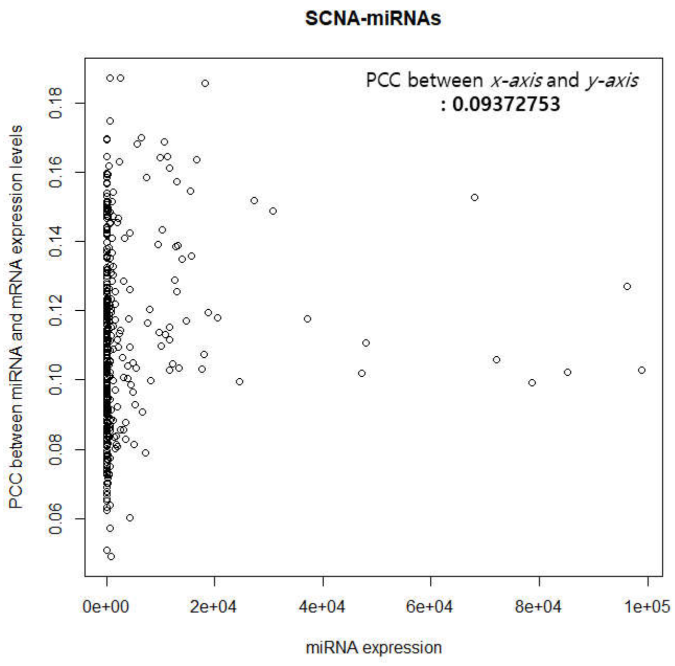

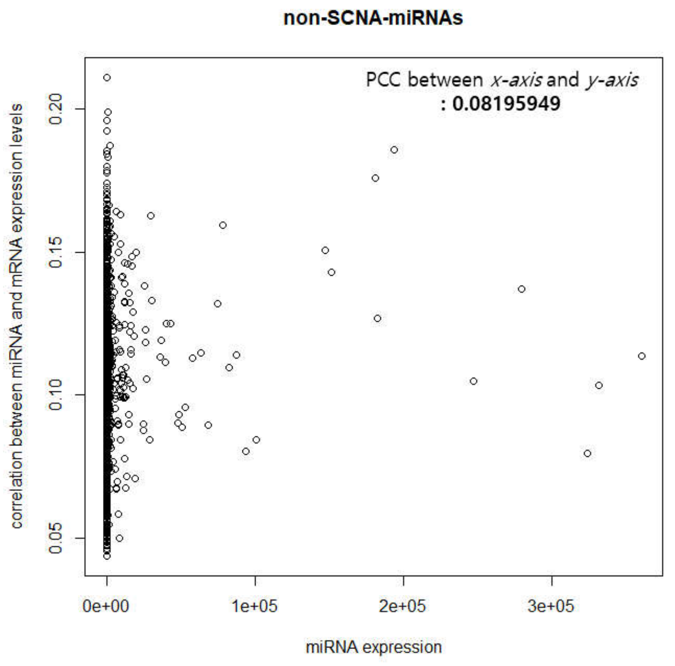

Median value of all miRNA expression value is 1.459. Scatter plots below represent the PCCs that miRNA expressions are lower than 1.459.

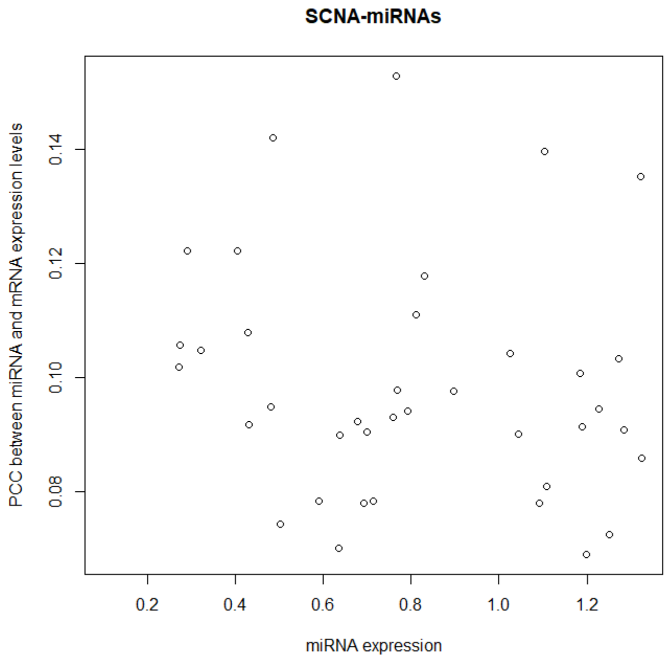
SCNA-miRNAs
PCC between miRNA and mRNA expression levels
0.08
0.10
0.12
0.14
0.2
0.4
0.6
0.8
1.0
1.2
miRNA expression

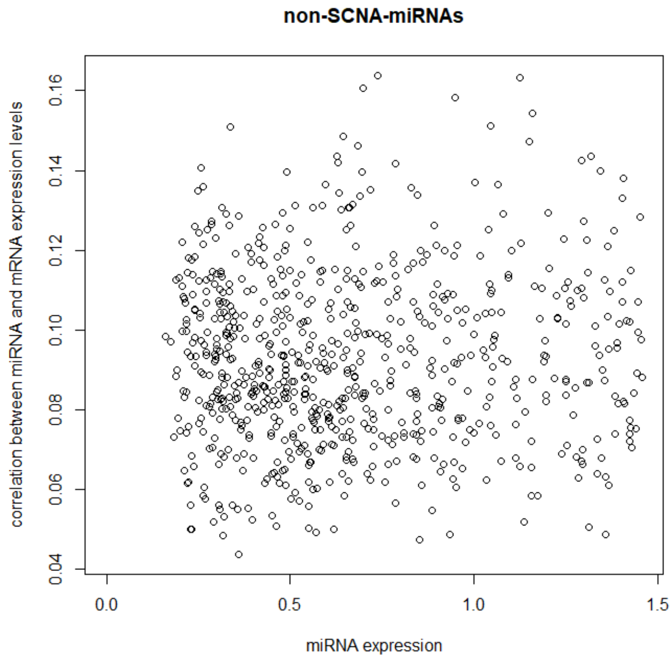
non-SCNA-miRNAs
correlation between miRNA and mRNA expression levels
0.04
0.06
0.08
0.10
0.12
0.14
0.16
0.0
0.5
1.0
1.5
miRNA expression

6. Supplementary Tables

6.1. Table S1

Analysis of patient survival according to copy numbers of the 84 pairs of SCNA-miRNAs and cancer types (Figure 5). The 32 pairs have significant q-value (<0.05). Results of the log rank test (p-value and q-value (Benjamini-Hochberg, BH) and cox regression (hazard ratio, HR) are shown.

Cancer	miRNA	p-value	q-value (BH)	HR	
BRCA	hsa-mir-106b	0.017682	0.094	2.365168	
BRCA	hsa-mir-15b	0.006459	0.068	2.44824	
BRCA	hsa-mir-16-2	0.004795	0.064	2.527681	
BRCA	hsa-mir-25	0.017682	0.094	2.365168	
BRCA	hsa-mir-3127	0.012141	0.094	4.047229	
BRCA	hsa-mir-33a	0.004397	0.064	3.667996	
BRCA	hsa-mir-3610	0.021807	0.096	1.937294	
BRCA	hsa-mir-489	0.000416	0.022	5.19958	
BRCA	hsa-mir-579	0.02047	0.096	2.315265	
BRCA	hsa-mir-580	0.001773	0.047	3.057062	
BRCA	hsa-mir-589	0.048402	0.18	2.043247	
BRCA	hsa-mir-624	0.03028	0.12	2.538413	
BRCA	hsa-mir-653	0.013142	0.094	2.556868	
BRCA	hsa-mir-93	0.017682	0.094	2.365168	
BRCA	hsa-let-7g	0.001913	0.011	2.515148	
BRCA	hsa-mir-101-2	0.000929	0.0071	2.923375	
BRCA	hsa-mir-1266	0.034931	0.072	1.851828	
BRCA	hsa-mir-138-1	0.007305	0.034	2.954905	
BRCA	hsa-mir-186	0.02952	0.068	2.02771	
BRCA	hsa-mir-197	0.037465	0.072	1.900833	
BRCA	hsa-mir-23b	0.01813	0.051	2.335126	
BRCA	hsa-mir-27b	0.01813	0.051	2.335126	
BRCA	hsa-mir-31	0.000281	0.0065	3.103074	
BRCA	hsa-mir-32	0.013627	0.051	2.341011	
BRCA	hsa-mir-491	0.00078	0.0071	2.950343	
BRCA	hsa-mir-628	0.019874	0.051	1.958414	
HNSC	hsa-mir-1301	0.04291	0.62	3.414339	Floor of mouth
HNSC	hsa-mir-3610	0.047346	0.62	2.955794	Floor of mouth
HNSC	hsa-mir-548s	0.017613	0.62	5.131651	Floor of mouth
HNSC	hsa-mir-339	0.001317	0.035	3.232167	Larynx
HNSC	hsa-mir-548s	9.11×10^{-6}	0.00048	22.06421	Larynx

HNSC	hsa-mir-589	0.00484	0.086	2.758341	Larynx
HNSC	hsa-mir-106b	0.025425	0.45	2.437404	Oral Cavity
HNSC	hsa-mir-25	0.025425	0.45	2.437404	Oral Cavity
HNSC	hsa-mir-93	0.025425	0.45	2.437404	Oral Cavity
HNSC	hsa-mir-548j	1.97×10^{-9}	4.50×10^{-8}	3.12×10^{16}	Larynx
HNSC	hsa-let-7g	0.035372	0.19	2.065728	Oral Cavity
HNSC	hsa-mir-138-1	0.043646	0.19	2.052824	Oral Cavity
HNSC	hsa-mir-186	2.26×10^{-8}	5.20×10^{-7}	64.49806	Oral Cavity
HNSC	hsa-mir-30a	0.006173	0.071	6.430202	Oral Cavity
HNSC	hsa-mir-320a	0.031323	0.19	2.095973	Oral Cavity
HNSC	hsa-mir-1180	0.028868	0.4	2.821504	Oral Tongue
HNSC	hsa-mir-1266	0.038118	0.4	2.538354	Oral Tongue
HNSC	hsa-mir-185	0.027257	0.11	5.267848	Floor of mouth
HNSC	hsa-mir-185	0.002828	0.011	16.09463	Larynx
HNSC	hsa-mir-23a	0.011671	0.023	5.66415	Oral Cavity
HNSC	hsa-mir-27a	0.011671	0.023	5.66415	Oral Cavity
KIRC	hsa-let-7i	0.00817	0.11	2.293408	
KIRC	hsa-mir-141	8.54×10^{-5}	0.0032	3.13531	
KIRC	hsa-mir-200c	0.000119	0.0032	3.060814	
KIRC	hsa-mir-3913-1	0.007745	0.11	2.313761	
KIRC	hsa-mir-944	0.01198	0.13	3.288119	
KIRC	hsa-mir-101-2	0.00749	0.029	2.11131	
KIRC	hsa-mir-1180	0.047031	0.087	2.458743	
KIRC	hsa-mir-146a	6.18×10^{-5}	0.00047	11.98799	
KIRC	hsa-mir-17	1.70×10^{-6}	2.10×10^{-5}	5.231795	
KIRC	hsa-mir-186	0.026408	0.055	2.520358	
KIRC	hsa-mir-1976	0.049386	0.087	1.913263	
KIRC	hsa-mir-19b-1	1.79×10^{-6}	2.10×10^{-5}	5.216482	
KIRC	hsa-mir-210	0.01781	0.044	7.884796	
KIRC	hsa-mir-23b	0.019267	0.044	1.930646	
KIRC	hsa-mir-27b	0.019267	0.044	1.930646	
KIRC	hsa-mir-31	0.00041	0.0024	2.616626	
KIRC	hsa-mir-32	0.012028	0.04	2.021522	
KIRC	hsa-mir-491	0.005043	0.023	2.227658	
LUAD	hsa-let-7i	0.044147	0.39	1.795552	
LUAD	hsa-mir-1224	0.030805	0.33	1.953468	
LUAD	hsa-mir-141	0.009351	0.17	2.181338	
LUAD	hsa-mir-200c	0.009351	0.17	2.181338	
LUAD	hsa-mir-33a	0.007819	0.17	3.316618	

LUAD	hsa-let-7g	0.046057	0.35	1.627937
LUAD	hsa-mir-138-1	0.032453	0.35	1.729908
LUAD	hsa-mir-320a	0.014983	0.34	1.678221
LUSC	hsa-mir-32	0.035008	0.51	1.764941
UCEC	hsa-mir-1248	0.045311	0.8	3.039579
UCEC	hsa-mir-339	0.001399	0.074	11.67031
UCEC	hsa-mir-570	0.021675	0.57	4.033702
UCEC	hsa-let-7g	0.002471	0.028	9.296557
UCEC	hsa-mir-138-1	0.001041	0.024	18.92333
UCEC	hsa-mir-186	0.005052	0.039	14.28257
UCEC	hsa-mir-197	0.032667	0.11	8.843942
UCEC	hsa-mir-23b	0.011504	0.049	3.947574
UCEC	hsa-mir-27b	0.011504	0.049	3.947574
UCEC	hsa-mir-32	0.012694	0.049	3.819131

6.2. Table S2

Comparison of survival times between the groups with SCNA-miRNAs and other miRNAs according to miRNA copy numbers.

Total p-value = 0.00254

	SCNA-miRNAs	Non- SCNA-miRNAs
Survival significant	30	118
Survival non-significant	530	4085
Total	560	4203

BLCA p-value = 1

	SCNA-miRNAs	Non- SCNA-miRNAs
Survival significant	0	2
Survival non-significant	80	548
Total	80	550

BRCA p-value = 1

	SCNA-miRNAs	Non- SCNA-miRNAs
Survival significant	6	47
Survival non-significant	74	570
Total	80	617

HNSC p-value = 0.000001345

	SCNA-miRNAs	Non- SCNA-miRNAs
Survival significant	7	1
Survival non-significant	73	627
Total	80	628

KIRC p-value = 0.09267

	SCNA-miRNAs	Non- SCNA-miRNAs
Survival significant	11	46
Survival non-significant	69	528
Total	80	574

LUAD p-value = 0.6084

	SCNA-miRNAs	Non- SCNA-miRNAs
Survival significant	0	9
Survival non-significant	80	596
Total	80	605

LUSC p-value = 1

	SCNA-miRNAs	Non- SCNA-miRNAs
Survival significant	0	6
Survival non-significant	80	613
Total	80	619

UCEC p-value = 0.001783

	SCNA-miRNAs	Non- SCNA-miRNAs
Survival significant	6	7
Survival non-significant	74	603
Total	80	610

6.3. Table S3

miRNAs commonly correlated with SCNAs across seven cancer types.

miRNA	BLCA	BRCA	HNSC	KIRC	LUAD	LUSC	UCEC	MULTIPLY	Log	Log*-2	*P*-value	Rank
hsa-mir-937	0.02	0.00	0.01	0.05	0.00	0.05	0.05	4.34E-13	-28.47	56.93	4.02E-07	1
hsa-mir-423	0.01	0.01	0.05	0.01	0.01	0.05	0.11	5.24E-13	-28.28	56.55	4.67E-07	2
hsa-mir-320a	0.04	0.01	0.01	0.06	0.01	0.12	0.01	3.10E-12	-26.50	53.00	1.90E-06	3
hsa-mir-28	0.06	0.02	0.00	0.05	0.06	0.01	0.03	3.29E-12	-26.44	52.88	1.99E-06	4
hsa-mir-30d	0.02	0.00	0.11	0.04	0.09	0.14	0.00	6.35E-12	-25.78	51.57	3.33E-06	5
hsa-mir-3913-1	0.00	0.02	0.02	0.24	0.00	0.08	0.32	6.72E-12	-25.73	51.45	3.48E-06	6
hsa-mir-15b	0.03	0.02	0.01	0.15	0.06	0.00	0.08	7.96E-12	-25.56	51.11	3.97E-06	7
hsa-mir-186	0.12	0.02	0.06	0.00	0.04	0.04	0.02	1.50E-11	-24.92	49.85	6.47E-06	8
hsa-mir-25	0.13	0.04	0.04	0.03	0.02	0.01	0.04	5.07E-11	-23.71	47.41	1.64E-05	9
hsa-mir-106b	0.08	0.06	0.02	0.01	0.03	0.04	0.08	8.03E-11	-23.25	46.49	2.33E-05	10
hsa-mir-589	0.02	0.23	0.08	0.03	0.00	0.08	0.04	8.83E-11	-23.15	46.30	2.50E-05	11
hsa-mir-548k	0.06	0.03	0.00	0.71	0.12	0.00	0.35	1.36E-10	-22.72	45.43	3.47E-05	12
hsa-mir-200c	0.14	0.01	0.01	0.23	0.06	0.01	0.14	2.09E-10	-22.29	44.58	4.77E-05	13
hsa-mir-1180	0.02	0.02	0.16	0.03	0.02	0.08	0.10	4.14E-10	-21.61	43.21	7.92E-05	14
hsa-mir-30e	0.03	0.03	0.12	0.23	0.04	0.07	0.01	5.31E-10	-21.36	42.71	9.52E-05	15
hsa-mir-182	0.16	0.03	0.06	0.12	0.01	0.03	0.07	5.40E-10	-21.34	42.68	9.65E-05	16
hsa-mir-141	0.21	0.01	0.03	0.19	0.03	0.01	0.19	8.72E-10	-20.86	41.72	1.37E-04	17
hsa-mir-96	0.12	0.04	0.06	0.16	0.01	0.03	0.07	9.72E-10	-20.75	41.50	1.48E-04	18
hsa-mir-183	0.16	0.04	0.06	0.15	0.01	0.03	0.06	1.36E-09	-20.42	40.84	1.89E-04	19
hsa-mir-491	0.01	0.06	0.07	0.33	0.02	0.12	0.03	1.50E-09	-20.32	40.64	2.03E-04	20
hsa-mir-92b	0.02	0.01	0.09	0.07	0.05	0.18	0.16	1.96E-09	-20.05	40.10	2.46E-04	21
hsa-mir-339	0.04	0.21	0.10	0.02	0.03	0.09	0.05	2.04E-09	-20.01	40.02	2.53E-04	22
hsa-mir-30b	0.06	0.01	0.15	0.06	0.12	0.21	0.03	2.48E-09	-19.82	39.63	2.91E-04	23
hsa-mir-3127	0.03	0.09	0.02	0.14	0.08	0.04	0.23	7.19E-09	-18.75	37.50	6.19E-04	24
hsa-mir-93	0.14	0.06	0.07	0.07	0.13	0.04	0.10	1.60E-08	-17.95	35.90	1.08E-03	25
hsa-mir-22	0.01	0.19	0.28	0.77	0.02	0.06	0.03	1.76E-08	-17.86	35.71	1.15E-03	26
hsa-mir-1301	0.08	0.09	0.15	0.07	0.15	0.02	0.09	2.10E-08	-17.68	35.36	1.30E-03	27
hsa-mir-197	0.14	0.10	0.12	0.11	0.06	0.07	0.03	3.04E-08	-17.31	34.62	1.67E-03	28
hsa-mir-1976	0.45	0.07	0.03	0.39	0.04	0.05	0.04	3.34E-08	-17.22	34.43	1.78E-03	29
hsa-mir-16-2	0.25	0.08	0.10	0.25	0.12	0.00	0.14	3.99E-08	-17.04	34.07	2.01E-03	30
hsa-mir-877	0.11	0.04	0.05	0.49	0.05	0.09	0.10	5.10E-08	-16.79	33.58	2.37E-03	31
hsa-mir-570	0.15	0.08	0.14	0.30	0.14	0.03	0.02	5.46E-08	-16.72	33.45	2.48E-03	32
hsa-mir-590	0.12	0.09	0.27	0.01	0.14	0.07	0.15	6.12E-08	-16.61	33.22	2.68E-03	33
hsa-mir-769	0.07	0.17	0.11	0.39	0.01	0.11	0.09	7.00E-08	-16.47	32.95	2.93E-03	34
hsa-mir-454	0.10	0.02	0.43	0.10	0.16	0.04	0.21	9.47E-08	-16.17	32.35	3.58E-03	35
hsa-mir-23a	0.40	0.24	0.21	0.53	0.22	0.03	0.00	9.70E-08	-16.15	32.30	3.63E-03	36
hsa-let-7g	0.01	0.29	0.04	0.26	0.32	0.29	0.05	1.09E-07	-16.04	32.07	3.91E-03	37
hsa-mir-31	0.00	0.47	0.01	0.64	0.23	0.07	0.72	1.17E-07	-15.96	31.92	4.11E-03	38
hsa-mir-1248	0.16	0.16	0.05	0.24	0.15	0.02	0.20	1.50E-07	-15.71	31.43	4.83E-03	39
hsa-mir-1266	0.73	0.09	0.26	0.62	0.04	0.10	0.00	1.52E-07	-15.70	31.40	4.87E-03	40
hsa-mir-32	0.05	0.29	0.10	0.06	0.09	0.05	0.43	1.78E-07	-15.54	31.08	5.40E-03	41

hsa-mir-580	0.01	0.11	0.14	0.75	0.04	0.10	0.42	1.90E-07	-15.48	30.95	5.63E-03	42
hsa-mir-185	0.07	0.04	0.23	0.35	0.05	0.04	0.52	2.03E-07	-15.41	30.82	5.88E-03	43
hsa-mir-19b-1	0.26	0.07	0.08	0.45	0.04	0.10	0.09	2.61E-07	-15.16	30.32	6.90E-03	44
hsa-mir-17	0.29	0.07	0.13	0.19	0.02	0.16	0.17	3.03E-07	-15.01	30.02	7.59E-03	45
hsa-mir-671	0.10	0.18	0.19	0.12	0.11	0.02	0.33	3.20E-07	-14.96	29.91	7.85E-03	46
hsa-mir-1306	0.07	0.11	0.14	0.32	0.12	0.03	0.27	3.61E-07	-14.83	29.67	8.47E-03	47
hsa-mir-27a	0.64	0.22	0.20	0.61	0.24	0.01	0.01	3.71E-07	-14.81	29.61	8.63E-03	48
hsa-mir-3682	0.11	0.05	0.11	0.22	0.07	0.09	0.49	4.14E-07	-14.70	29.40	9.23E-03	49
hsa-mir-148b	0.04	0.39	0.35	0.27	0.08	0.07	0.06	4.93E-07	-14.52	29.05	1.03E-02	50
hsa-mir-101-2	0.24	0.27	0.03	0.10	0.18	0.09	0.17	5.86E-07	-14.35	28.70	1.15E-02	51
hsa-mir-7-1	0.13	0.13	0.02	0.18	0.19	0.42	0.19	7.39E-07	-14.12	28.24	1.32E-02	52
hsa-mir-27b	0.22	0.24	0.17	0.01	0.22	0.18	0.48	9.73E-07	-13.84	27.69	1.57E-02	53
hsa-mir-624	0.28	0.40	0.09	0.18	0.03	0.15	0.12	9.81E-07	-13.83	27.67	1.57E-02	54
hsa-mir-301a	0.13	0.05	0.42	0.14	0.13	0.08	0.22	9.87E-07	-13.83	27.66	1.58E-02	55
hsa-mir-24-1	0.06	0.32	0.05	0.05	0.21	0.17	0.61	1.08E-06	-13.74	27.47	1.67E-02	56
hsa-mir-579	0.03	0.29	0.29	0.39	0.09	0.02	0.58	1.25E-06	-13.59	27.19	1.82E-02	57
hsa-mir-744	0.09	0.11	0.21	0.11	0.15	0.18	0.22	1.36E-06	-13.51	27.01	1.92E-02	58
hsa-mir-330	0.08	0.23	0.20	0.73	0.03	0.13	0.16	1.40E-06	-13.48	26.96	1.95E-02	59
hsa-mir-26b	0.23	0.18	0.29	0.02	0.55	0.07	0.14	1.57E-06	-13.37	26.73	2.08E-02	60
hsa-mir-3664	0.18	0.12	0.02	0.76	0.17	0.09	0.36	1.74E-06	-13.26	26.53	2.22E-02	61
hsa-mir-1224	0.09	0.27	0.01	0.48	0.50	0.06	0.67	1.92E-06	-13.17	26.33	2.35E-02	62
hsa-let-7d	0.41	0.40	0.03	0.14	0.09	0.09	0.34	2.09E-06	-13.08	26.16	2.47E-02	63
hsa-mir-1237	0.07	0.20	0.87	0.03	0.27	0.17	0.16	2.38E-06	-12.95	25.89	2.67E-02	64
hsa-mir-335	0.13	0.36	0.14	0.27	0.08	0.12	0.17	2.52E-06	-12.89	25.78	2.76E-02	65
hsa-mir-30c-1	0.05	0.10	0.19	0.37	0.24	0.30	0.11	3.01E-06	-12.71	25.43	3.06E-02	66
hsa-mir-3074	0.11	0.53	0.19	0.11	0.21	0.12	0.10	3.24E-06	-12.64	25.28	3.19E-02	67
hsa-mir-550a-2	0.06	0.22	0.28	0.26	0.10	0.27	0.15	3.61E-06	-12.53	25.06	3.40E-02	68
hsa-mir-23b	0.33	0.24	0.16	0.02	0.25	0.14	0.44	4.02E-06	-12.42	24.85	3.61E-02	69
hsa-mir-3125	0.17	0.31	0.04	0.08	0.30	0.11	0.82	4.06E-06	-12.41	24.83	3.63E-02	70
hsa-mir-942	0.19	0.12	0.06	0.40	0.38	0.28	0.07	4.26E-06	-12.37	24.73	3.73E-02	71
hsa-mir-3610	0.12	0.03	0.53	0.41	0.25	0.21	0.13	4.34E-06	-12.35	24.69	3.77E-02	72
hsa-mir-130b	0.09	0.16	0.14	0.39	0.18	0.06	0.59	4.90E-06	-12.23	24.45	4.04E-02	73
hsa-let-7b	0.31	0.14	0.12	0.14	0.19	0.31	0.13	5.17E-06	-12.17	24.34	4.16E-02	74
hsa-mir-324	0.12	0.30	0.08	0.32	0.19	0.25	0.15	6.94E-06	-11.88	23.76	4.90E-02	75
hsa-mir-331	0.08	0.27	0.21	0.18	0.38	0.12	0.18	6.97E-06	-11.87	23.75	4.91E-02	76

6.4. Table S4

Univariate analysis of cancers. Variables differ depending on cancer types.

	beta	HR (95% CI for HR)	wald.test	p.value
**gender**	-0.054	0.95 (0.48-1.9)	0.02	0.88
**history_other_malignancy**	-0.28	0.76 (0.41-1.4)	0.77	0.38
**pathologic_T**	0.39	1.5 (1.1-2.1)	5.2	0.023
**pathologic_N**	0.38	1.5 (1.1-2)	6.8	0.0094
**pathologic_M**	-0.29	0.75 (0.1-5.4)	0.08	0.77
**pathologic_stage**	0.56	1.8 (1.2-2.6)	8.9	0.0029
**age_at_initial_pathologic_diagnosis (<60, >=60)**	0.016	1 (0.99-1)	1.1	0.3
**number_pack_years_smoked (<30, >=30)**	0.0056	1 (1-1)	1.2	0.27

BLCA

	beta	HR (95% CI for HR)	wald.test	p.value
**history_other_malignancy**	0.69	2 (0.72-5.5)	1.8	0.19
**pathologic_T**	0.22	1.2 (0.92-1.7)	2	0.16
**pathologic_N**	0.57	1.8 (1.3-2.3)	16	5.60E-05
**pathologic_M**	1.3	3.6 (0.87-15)	3.2	0.076
**pathologic_stage**	0.56	1.7 (1.2-2.5)	9.4	0.0022
**breast_carcinoma_estrogen_receptor_status**	-0.29	0.75 (0.43-1.3)	1.1	0.3
**breast_carcinoma_progesterone_receptor_status**	-0.14	0.87 (0.52-1.5)	0.28	0.59
**age_at_initial_pathologic_diagnosis (<60, >=60)**	0.021	1 (1-1)	4.8	0.028

BRCA

HNSC

	beta	HR (95% CI for HR)	wald.test	p.value
**gender**	0.19	1.2 (0.85-1.7)	1.1	0.29
**history_other_malignancy**	-0.44	0.64 (0.26-1.6)	0.94	0.33
**pathologic_T**	0.19	1.2 (1.1-1.4)	8.3	0.004
**pathologic_N**	0.25	1.3 (1.1-1.5)	8	0.0047
**pathologic_stage**	0.17	1.2 (1-1.3)	6.8	0.0089
**alcohol_history_documented (yes, no)**	-0.27	0.76 (0.55-1.1)	2.6	0.11
**age_at_initial_pathologic_diagnosis (<60, >=60)**	0.024	1 (1-1)	9.9	0.0016
**number_pack_years_smoked (<30, >=30)**	-0.00052	1 (0.99-1)	0.03	0.86

KIRC

	beta	HR (95% CI for HR)	wald.test	p.value
**gender**	0.06	1.1 (0.64-1.8)	0.05	0.82
**history_other_malignancy**	0.097	1.1 (0.57-2.1)	0.08	0.78
**pathologic_T**	0.17	1.2 (0.88-1.6)	1.3	0.26
**pathologic_N**	0.074	1.1 (0.79-1.5)	0.22	0.64
**pathologic_M**	1.6	4.9 (1.2-20)	4.9	0.027
**pathologic_stage**	0.11	1.1 (0.85-1.5)	0.67	0.41
**age_at_initial_pathologic_diagnosis (<60, >=60)**	0.0045	1 (0.97-1)	0.09	0.77
**number_pack_years_smoked (<30, >=30)**	-0.0055	0.99 (0.99-1)	2.6	0.11

	beta	HR (95% CI for HR)	wald.test	p.value
**gender**	0.1	1.1 (0.66-1.9)	0.14	0.71
**history_other_malignancy**	0.039	1 (0.56-1.9)	0.01	0.9
**pathologic_T**	0.67	2 (1.5-2.5)	25	6.00E-07
**pathologic_N**	1.9	6.6 (2.6-17)	16	6.50E-05
**pathologic_M**	1.5	4.3 (2.6-7)	34	4.30E-09
**pathologic_stage**	0.67	2 (1.6-2.4)	38	6.40E-10
**age_at_initial_pathologic_diagnosis (<60, >=60)**	0.029	1 (1-1)	8.1	0.0044

LUAD

	beta	HR (95% CI for HR)	wald.test	p.value
**gender**	0.072	1.1 (0.72-1.6)	0.12	0.73
**history_other_malignancy**	0.5	1.6 (0.94-2.9)	3	0.084
**pathologic_T**	0.35	1.4 (1.1-1.8)	7	0.0081
**pathologic_N**	0.56	1.8 (1.4-2.2)	20	6.00E-06
**pathologic_M**	0.34	1.4 (0.68-2.9)	0.82	0.37
**pathologic_stage**	0.41	1.5 (1.3-1.8)	19	1.40E-05
**age_at_initial_pathologic_diagnosis (<60, >=60)**	-0.016	0.98 (0.97-1)	2.7	0.1
**number_pack_years_smoked (<30, >=30)**	-0.0029	1 (0.99-1)	0.55	0.46

LUSC

UCEC

	beta	HR (95% CI for HR)	wald.test	p.value
**age_at_initial_pathologic_diagnosis (<60, >=60)**	0.0086	1 (0.97-1)	0.2	0.66

6.5. Table S5

Multivariate analysis of miRNAs as prognostic factors based on copy number state.

[BRCA]

Let-7g

variable	HR (95%CI)	P
history_other_malignancy	3.18 (1.09-9.3)	0.034
pathologic_T	0.94 (0.62-1.41)	0.76
pathologic_N	1.67 (1.11-2.52)	0.015
pathologic_M	1.13 (0.22-5.89)	0.886
pathologic_stage	1.21 (0.68-2.16)	0.507
breast_carcinoma_estrogen_receptor_status	0.72 (0.28-1.81)	0.484
breast_carcinoma_progesterone_receptor_status	1.18 (0.48-2.92)	0.714
age_at_initial_pathologic_diagnosis	1.03 (1.01-1.05)	0.001
Copy number state	3.49 (1.82-6.7)	0

Mir-101-2

variable	HR (95%CI)	P
[illegible]	[illegible]	[illegible]

variable	HR (95%CI)	P
history_other_malignancy	2.2 (0.27-17.54)	0.458
pathologic_T	0.98 (0.6-1.62)	0.946
pathologic_N	2.36 (1.5-3.72)	0
pathologic_M	0 (0-Inf)	0.998
pathologic_stage	1.27 (0.7-2.32)	0.431
breast_carcinoma_estrogen_receptor_status	0.17 (0.05-0.58)	0.005
breast_carcinoma_progesterone_receptor_status	3.28 (1.05-10.22)	0.04
age_at_initial_pathologic_diagnosis	1.02 (0.99-1.04)	0.268
Copy number state	3.37 (1.4-8.13)	0.007

Mir-138-1

Mir-31

variable	HR (95%CI)	P
history_other_malignancy	3.58 (1.2-10.64)	0.022
pathologic_T	0.99 (0.59-1.66)	0.962
pathologic_N	1.54 (0.92-2.58)	0.1
pathologic_M	1.11 (0.2-6.01)	0.904
pathologic_stage	1.23 (0.52-2.91)	0.64
breast_carcinoma_estrogen_receptor_status	0.98 (0.38-2.54)	0.973
breast_carcinoma_progesterone_receptor_status	0.71 (0.3-1.68)	0.439
age_at_initial_pathologic_diagnosis	1.04 (1.01-1.06)	0.001
Copy number state	3.46 (1.7-7.06)	0.001

ir-489

variable	HR (95%CI)	P
history_other_malignancy	4.06 (1.04-15.8)	0.043
pathologic_T	0.91 (0.45-1.85)	0.797
pathologic_N	2.17 (1.08-4.37)	0.03
pathologic_M	0.9 (0.09-9.49)	0.931
pathologic_stage	0.82 (0.26-2.58)	0.73
breast_carcinoma_estrogen_receptor_status	0.47 (0.11-2.06)	0.314
breast_carcinoma_progesterone_receptor_status	2.35 (0.47-11.84)	0.299
age_at_initial_pathologic_diagnosis	1.03 (1-1.07)	0.032
Copy number state	6.22 (1.62-23.81)	0.008

Mir-491

variable	HR (95%CI)	P
history_other_malignancy	3.58 (1.2-10.65)	0.022
pathologic_T	0.99 (0.59-1.68)	0.977
pathologic_N	1.54 (0.92-2.59)	0.104
pathologic_M	1.19 (0.22-6.5)	0.837
pathologic_stage	1.18 (0.49-2.83)	0.713
breast_carcinoma_estrogen_receptor_status	0.89 (0.33-2.39)	0.813
breast_carcinoma_progesterone_receptor_status	0.79 (0.32-1.96)	0.612
age_at_initial_pathologic_diagnosis	1.04 (1.02-1.06)	0.001
Copy number state	3.38 (1.63-6.99)	0.001

Mir-580

variable	HR (95%CI)	P
history_other_malignancy	0 (0-Inf)	0.997
pathologic_T	1.24 (0.74-2.07)	0.422
pathologic_N	1.92 (1.13-3.24)	0.015
pathologic_M	0 (0-Inf)	1
pathologic_stage	0.8 (0.48-1.34)	0.402
breast_carcinoma_estrogen_receptor_status	2.48 (0.78-7.86)	0.122
breast_carcinoma_progesterone_receptor_status	0.57 (0.2-1.57)	0.275
age_at_initial_pathologic_diagnosis	1.03 (1-1.05)	0.057
Copy number state	3.88 (1.63-9.24)	0.002

[HNSC]

Mir-185

variable	HR (95%CI)	P
gender	2.53 (0.91-7.04)	0.076
history_other_malignancy	0 (0-Inf)	0.997
pathologic_T	1.26 (0.45-3.53)	0.661
pathologic_N	0.95 (0.57-1.58)	0.842
pathologic_M	NA (NA-NA)	NA
pathologic_stage	0.7 (0.23-2.12)	0.523
alcohol_history_documented	0.63 (0.29-1.35)	0.234
age_at_initial_pathologic_diagnosis	1.01 (0.96-1.05)	0.83
number_pack_years_smoked	1 (0.98-1.01)	0.425
Copy number state	14.36 (1.08-190.8)	0.044

Mir-186

variable	HR (95%CI)	P
gender	0.48 (0.18-1.26)	0.136
history_other_malignancy	5.22 (0.59-46.29)	0.138
pathologic_T	0.69 (0.24-1.98)	0.494
pathologic_N	0.82 (0.49-1.4)	0.472
pathologic_M	NA (NA-NA)	NA
pathologic_stage	2.91 (0.9-9.44)	0.075
alcohol_history_documented	0.52 (0.21-1.27)	0.152
age_at_initial_pathologic_diagnosis	1.03 (0.99-1.07)	0.155
number_pack_years_smoked	1.01 (0.99-1.02)	0.433
Copy number state	28.84 (1.27-657.29)	0.035

Mir-23a

variable	HR (95%CI)	P
gender	0.43 (0.16-1.15)	0.093
history_other_malignancy	4.86 (0.53-44.52)	0.162
pathologic_T	0.95 (0.31-2.91)	0.929
pathologic_N	0.93 (0.53-1.61)	0.791
pathologic_M	NA (NA-NA)	NA
pathologic_stage	1.95 (0.57-6.65)	0.285
alcohol_history_documented	0.62 (0.26-1.46)	0.27
age_at_initial_pathologic_diagnosis	1.04 (1-1.08)	0.045
number_pack_years_smoked	1 (0.98-1.01)	0.673
Copy number state	5.96 (1.01-35.04)	0.048

Mir-27a

variable	HR (95%CI)	P
gender	0.43 (0.16-1.15)	0.093
history_other_malignancy	4.86 (0.53-44.52)	0.162
pathologic_T	0.95 (0.31-2.91)	0.929
pathologic_N	0.93 (0.53-1.61)	0.791
pathologic_M	NA (NA-NA)	NA
pathologic_stage	1.95 (0.57-6.65)	0.285
alcohol_history_documented	0.62 (0.26-1.46)	0.27
age_at_initial_pathologic_diagnosis	1.04 (1-1.08)	0.045
number_pack_years_smoked	1 (0.98-1.01)	0.673
Copy number state	5.96 (1.01-35.04)	0.048

Mir-339

variable	HR (95%CI)	P
[illegible]	[illegible]	[illegible]

variable	HR (95%CI)	P
gender	1.83 (0.28-11.74)	0.525
history_other_malignancy	0 (0-Inf)	0.998
pathologic_T	1.56 (0.36-6.72)	0.549
pathologic_N	1.06 (0.48-2.36)	0.878
pathologic_M	1 (1-1)	NA
pathologic_stage	0.48 (0.07-3.08)	0.438
alcohol_history_documented	0.61 (0.18-2.11)	0.433
age_at_initial_pathologic_diagnosis	0.98 (0.91-1.05)	0.549
number_pack_years_smoked	1.02 (0.99-1.05)	0.178
Copy number state	58 (5.45-617.49)	0.001

Mir-548s

[KIRC]

variable	HR (95%CI)	P
gender	1.02 (0.59-1.78)	0.936
history_other_malignancy	2.48 (1.12-5.51)	0.025
pathologic_T	0.63 (0.37-1.06)	0.08
pathologic_N	4.76 (1.71-13.22)	0.003
pathologic_M	0.49 (0.2-1.22)	0.126
pathologic_stage	3.42 (1.9-6.16)	0
age_at_initial_pathologic_diagnosis	1.02 (1-1.05)	0.072
Copy number state	1.81 (1.01-3.25)	0.046

Mir-101-2
Mir-17

variable	HR (95%CI)	P
gender	1.07 (0.61-1.88)	0.819
history_other_malignancy	3.05 (1.37-6.77)	0.006
pathologic_T	0.53 (0.32-0.9)	0.018
pathologic_N	5.24 (1.91-14.4)	0.001
pathologic_M	0.65 (0.26-1.61)	0.347
pathologic_stage	3.68 (2.03-6.69)	0
age_at_initial_pathologic_diagnosis	1.02 (0.99-1.04)	0.216
Copy number state	5.69 (2.52-12.84)	0

ir-19b-1

variable	HR (95%CI)	P
gender	1.07 (0.61-1.88)	0.816
history_other_malignancy	3.04 (1.37-6.76)	0.006
pathologic_T	0.53 (0.32-0.9)	0.018
pathologic_N	5.24 (1.91-14.39)	0.001
pathologic_M	0.65 (0.26-1.61)	0.348
pathologic_stage	3.68 (2.03-6.68)	0
age_at_initial_pathologic_diagnosis	1.02 (0.99-1.04)	0.216
Copy number state	5.69 (2.52-12.82)	0

Mir-31

variable	HR (95%CI)	P
gender	0.88 (0.49-1.57)	0.657
history_other_malignancy	2.73 (1.23-6.09)	0.014
pathologic_T	0.65 (0.39-1.09)	0.099
pathologic_N	4.6 (1.66-12.76)	0.003
pathologic_M	0.5 (0.2-1.24)	0.133
pathologic_stage	3.36 (1.85-6.08)	0
age_at_initial_pathologic_diagnosis	1.03 (1-1.05)	0.042
Copy number state	1.97 (1.11-3.49)	0.021

Mir-491

variable	HR (95%CI)	P
gender	1.03 (0.58-1.85)	0.913
history_other_malignancy	2.11 (0.9-4.93)	0.084
pathologic_T	0.66 (0.37-1.18)	0.159
pathologic_N	4.19 (1.47-11.92)	0.007
pathologic_M	0.6 (0.23-1.54)	0.29
pathologic_stage	3.08 (1.64-5.79)	0
age_at_initial_pathologic_diagnosis	1.03 (1-1.05)	0.035
Copy number state	2.02 (1.11-3.68)	0.022

[UCEC]

Let-7g

variable	HR (95%CI)	P
age_at_initial_pathologic_diagnosis	1.01 (0.97-1.05)	0.712
Copy number state	8.63 (1.5-49.69)	0.016

Mir-138-1

variable	HR (95%CI)	P
age_at_initial_pathologic_diagnosis	1.01 (0.96-1.06)	0.706
Copy number state	17.46 (1.53-198.6)	0.021

Mir-186

variable	HR (95%CI)	P
age_at_initial_pathologic_diagnosis	1.02 (0.97-1.06)	0.446
Copy number state	15.59 (1.29-188.14)	0.031

Mir-23b

variable	HR (95%CI)	P
age_at_initial_pathologic_diagnosis	1 (0.96-1.04)	0.885
Copy number state	4.03 (1.2-13.56)	0.024

Mir-27b

variable	HR (95%CI)	P
age_at_initial_pathologic_diagnosis	1 (0.96-1.04)	0.885
Copy number state	4.03 (1.2-13.56)	0.024

Mir-32

variable	HR (95%CI)	P
age_at_initial_pathologic_diagnosis	1.01 (0.96-1.05)	0.787
Copy number state	3.73 (1.19-11.65)	0.023

6.6. Table S6

Cancer-related miRNAs according to the HMDD. The HMDD contains 356 miRNAs relevant for the seven cancer types.

		BLCA	BRCA	HNSC	KIRC	LUAD	LUSC	UCEC
# of miRNAs for all seven cancers in HMDD (HMDD_all)		356						
# of miRNAs for each cancer type in HMDD (HMDD_ each cancer type)		106	243	97	135	194	157	110
# of CNV-miRNAs		80						
CNV-miRNAs	Intersection of HMDD_all and CNV-miRNAs	51						
	Intersection of HMDD_each cancer type and CNV-miRNAs	21	36	22	22	37	35	22
# of cancer type-specific CNV-miRNAs (CS-CNV-miRNAs)		21	23	15	11	22	44	24
CS-CNV-miRNAs	Intersection of HMDD_all and CS-CNV-miRNAs	10	16	13	9	13	26	17
	Intersection of HMDD_each cancer type and CS-CNV-miRNAs	2	10	8	5	7	17	4

6.7. Table S7

SCNA-miRNAs reported in HMDD and the results of literature search demonstrating SCNA-miRNA associations with cancer development and progression.

Rank	miRNA	HMDD	PMID		Evidence	
29	hsa-mir-1976	X		27063799	MicroRNA-1976 functions as a tumor suppressor and serves as a prognostic indicator in non-small cell lung cancer by directly targeting PLCE1.	Chen et al.
15	hsa-mir-30e	O				
8	hsa-mir-186	O				
28	hsa-mir-197	O				
21	hsa-mir-92b	X		27430302	miR-92b targets DAB2IP to promote EMT in bladder cancer migration and invasion.	Huang et al.
				26878388	Identification of differentially expressed miRNAs in individual breast cancer patient and application in personalized medicine.	Peng et al.
				24162673	Inhibition of miR-92b suppresses nonsmall cell lung cancer cells growth and motility by targeting RECK.	Lei et al.
148	hsa-mir-205	O				
93	hsa-mir-548s	X				
27	hsa-mir-1301	X		22159405	microRNA-1301-mediated inhibition of tumorigenesis.	Fang et al.
24	hsa-mir-3127	X		25849943	MicroRNA-3127 promotes cell proliferation and tumorigenicity in hepatocellular carcinoma by disrupting of PI3K/AKT negative regulation.	Jiangn et al.
				25284075	Reduced miR-3127-5p expression promotes NSCLC proliferation/invasion and contributes to dasatinib sensitivity via the c-Abl/Ras/ERK pathway.	Sun et al.
60	hsa-mir-26b	O				
215	hsa-mir-138-1	O				
37	hsa-let-7g	O				
7	hsa-mir-15b	O				
30	hsa-mir-16-2	O				
62	hsa-mir-1224	O				
39	hsa-mir-1248	X		24997136	MicroRNA-binding site SNPs in deregulated genes are associated with clinical outcome of non-small cell lung cancer.	Sun et al.
4	hsa-mir-28	O				
90	hsa-mir-944	O				
32	hsa-mir-570	X		26045791	MicroRNA-570 promotes lung carcinoma proliferation through targeting tumor suppressor KLF9.	Tong et al.
57	hsa-mir-579	X		27092493	Prognostic Value of MicroRNAs in Preoperative Treated Rectal Cancer.	Azizian et al.
42	hsa-mir-580	X		22952847	Consensus micro RNAs governing the switch of dormant tumors to the fast-growing angiogenic phenotype.	Almog et al.
119	hsa-mir-146a	O				
31	hsa-mir-877	X		27429046	Up-regulation of p16 by miR-877-3p inhibits proliferation of bladder cancer.	Li et al.
171	hsa-mir-30a	O				
22	hsa-mir-339	O				

11	hsa-mir-589	X		27835990	miR-589-5p inhibits MAP3K8 and suppresses CD90+ cancer stem cells in hepatocellular carcinoma.	Zhang et al.
33	hsa-mir-590	X		28012926	miR-590 accelerates lung adenocarcinoma migration and invasion through directly suppressing functional target OLFM4.	Liu et al.
				27770372	microRNA-590 suppresses the tumorigenesis and invasiveness of non-small cell lung cancer cells by targeting ADAM9.	Wang et al.
				24063284	Enhancement of proliferation and invasion by MicroRNA-590-5p via targeting PBRM1 in clear cell renal carcinoma cells.	Xiao et al.
172	hsa-mir-653	X				
161	hsa-mir-489	X		27216745	MicroRNA in late lung development and bronchopulmonary dysplasia: the need to demonstrate causality.	Nardiello et al.
				25833694	Down-regulation of miR-489 contributes into NSCLC cell invasion through targeting SUZ12.	Xie et al.
				20700123	miR-489 is a tumour-suppressive miRNA target PTPN11 in hypopharyngeal squamous cell carcinoma (HSCC).	Kikkawa et al.
9	hsa-mir-25	O				
25	hsa-mir-93	O				
10	hsa-mir-106b	O				
16	hsa-mir-182	O				
18	hsa-mir-96	O				
19	hsa-mir-183	O				
138	hsa-mir-29a	O				
46	hsa-mir-671	X		28035073	MicroRNA expression profiles and clinicopathological implications in lung adenocarcinoma according to EGFR, KRAS, and ALK status.	Kim et al.
3	hsa-mir-320a	O				
72	hsa-mir-3610	X				
23	hsa-mir-30b	O				
5	hsa-mir-30d	O				
1	hsa-mir-937	X		27179609	miR-937 contributes to the lung cancer cell proliferation by targeting INPP4B.	Zhang et al.
51	hsa-mir-101-2	O				
20	hsa-mir-491	O				
38	hsa-mir-31	O				
52	hsa-mir-7-1	O				
63	hsa-let-7d	O				
69	hsa-mir-23b	O				
53	hsa-mir-27b	O				
41	hsa-mir-32	O				
168	hsa-mir-210	O				
12	hsa-mir-548k	X		25086664	Multi-tiered genomic analysis of head and neck cancer ties TP53 mutation to 3p loss.	Gross et al.
13	hsa-mir-200c	O				
17	hsa-mir-141	O				

134	hsa-let-7i	O				
6	hsa-mir-3913-1	X				
45	hsa-mir-17	O				
44	hsa-mir-19b-1	O				
54	hsa-mir-624	X		23445303	Deregulation of glycolysis in cancer: glyceraldehyde-3-phosphate dehydrogenase as a therapeutic target.	Krasnov et al.
40	hsa-mir-1266	X		25640367	Association of miR-1266 with recurrence/metastasis potential in estrogen receptor positive breast cancer patients.	Sevinc et al.
				24481448	miR-1207-5p and miR-1266 suppress gastric cancer growth and invasion by targeting telomerase reverse transcriptase.	Chen et al.
185	hsa-mir-628	X		24477576	Circulatory miR-628-5p is downregulated in prostate cancer patients.	Favreau et al.
26	hsa-mir-22	O				
14	hsa-mir-1180	X		27112784	The suppressive effects of miR-1180-5p on the proliferation and tumorigenicity of bladder cancer cells.	Ge et al.
				27044843	MiR-1180 promoted the proliferation of hepatocellular carcinoma cells by repressing TNIP2 expression.	Zhou et al.
2	hsa-mir-423	O				
35	hsa-mir-454	O				
99	hsa-mir-3615	X				
137	hsa-mir-24-2	O				
48	hsa-mir-27a	O				
36	hsa-mir-23a	O				
82	hsa-mir-181c	O				
96	hsa-mir-181d	O				
113	hsa-mir-641	X		23732000	Exploration of tumor-suppressive microRNAs silenced by DNA hypermethylation in cervical cancer.	Yao et al.
					Abstract B28: MicroRNA-641 activates MAPK by targeting NF1 and cooperates with its host gene AKT2 in human cancer.	Richards et al.
117	hsa-mir-643	X		25722110	Identifying microRNA-mRNA regulatory network in gemcitabine-resistant cells derived from human pancreatic cancer cells.	Shen et al.
					Integration of mRNA and microRNA profiles as prognostic and predictive markers in lung adenocarcinoma.	Strickler et al.
84	hsa-mir-296	O				
43	hsa-mir-185	O				
47	hsa-mir-1306	X				
73	hsa-mir-130b	O				
109	hsa-mir-548j	X		26949125	MicroRNA-548j functions as a metastasis promoter in human breast cancer by targeting Tensin1.	Zhan et al.
174	hsa-mir-3200	X		26293775	Identification and characterization of microRNAs expressed in human breast cancer chemo-resistant MCF-7/Adr cells by Solexa deep-sequencing technology.	Xu et al.
98	hsa-mir-33a	O				

7. References

1) Calin, G. A.et al. Human microRNA genes are frequently located at fragile sites and genomic regions involved in cancers. Proceedings of the National Academy of Sciences101, 2999–3004 (2004).
2) Zhang, L.et al. microRNAs exhibit high frequency genomic alterations in human cancer. Proceedings of the National Academy of Sciences 103, 9136–9141 (2006).
3) Lamy, P. et al. Are microRNAs located in genomic regions associated with cancer? British Journal of Cancer 95, 1415–1418 (2006).
4) Wang, B. et al. MicroRNA-320a inhibits proliferation and invasion of breast cancer cells by targeting RAB11A. American Journal of Cancer Research 5, 2719 (2015).
5) Ling, B., Wang, G.-X., Long, G., Qiu, J.-H. & Hu, Z.-L. Tumor suppressor miR-22 suppresses lung cancer cell515progression through post-transcriptional regulation of ErbB3.Journal of Cancer Research and Clinical Oncology 516138, 1355–1361 (2012).
6) Summerer, I. et al. Circulating microRNAs as prognostic therapy biomarkers in head and neck cancer patients. British Journal Of Cancer 113, 76–82 (2015).
7) Aushev, V. N. et al. Comparisons of microRNA Patterns in Plasma before and after Tumor Removal Reveal New Biomarkers of Lung Squamous Cell Carcinoma. PLOS ONE 8, e78649 (2013).
8) Li, J. et al. Inhibition of miR-15b decreases cell migration and metastasis in colorectal cancer. Tumor Biology 37, 8765–8773 (2016).
9) Armstrong, D. A., Green, B. B., Seigne, J. D., Schned, A. R. & Marsit, C. J. MicroRNA molecular profiling from matched tumor and bio-fluids in bladder cancer. Molecular Cancer 14, 194 (2015).
10) Sakurai, M. et al. Correlation of miRNA Expression Profiling in Surgical Pathology Materials, with Ki-67, HER2, ER and PR in Breast Cancer Patients, The International Journal of Biological Markers 30, e190–9 (2014).
11) Shiiba, M., Uzawa, K. & Tanzawa, H. MicroRNAs in Head and Neck Squamous Cell Carcinoma (HNSCC) and Oral Squamous Cell Carcinoma (OSCC). Cancers 2, 653–669 (2010).
12) Aherne, S. T. et al. ltered expression of mir-222 and mir-25 influences diverse gene expression changes in transformed normal and anaplastic thyroid cells, and impacts on MEK and TRAIL protein expression. International Journal of Molecular Medicine 38, 433–445 (2016).
13) Wang, Z. et al. MicroRNA-25 regulates chemoresistance-associated autophagy in breast cancer cells, a process modulated by the natural autophagy inducer isoliquiritigenin. Oncotarget 5, 7013–7026 (2014).
14) Wu, T. et al. miR-25 targets the modulator of apoptosis 1 gene in lung cancer. Carcinogenesis 36, 925–935 (2015).
15) Molina-Pinelo, S. et al. MicroRNA clusters: dysregulation in lung adenocarcinoma and COPD. European Respiratory Journal 43, 1740–1749 (2014).
16) Hui, A. B. et al. Comprehensive MicroRNA Profiling for Head and Neck Squamous Cell Carcinomas. Clinical Cancer Research 16, 1129–1139 (2010).
17) Tejero, R.et al. miR-141 and miR-200c as Markers of Overall Survival in Early Stage Non-Small Cell Lung Cancer Adenocarcinoma. PLOS ONE 9, e101899 (2014).
18) Yang, M., Yao, Y., Eades, G., Zhang, Y. & Zhou, Q. MiR-28 regulates Nrf2 expression through a Keap1-independent mechanism. Breast Cancer Research and Treatment 129, 983–991 (2011).
19) D’aiuto, F. et al. miR-30e* is an independent subtype-specific prognostic marker in breast cancer. British Journal of Cancer 113, 290–298 (2015).
20) Gao, W. et al. MiR-21 overexpression in human primary squamous cell lung carcinoma is associated with poor patient prognosis. Journal of Cancer Research and Clinical Oncology 137, 557–566 (2011).

21) Ratner, E. S. et al. MicroRNA signatures differentiate uterine cancer tumor subtypes. Gynecologic Oncology 118, 251–257 (2010).
22) Wang, G. et al. Up-regulation of microRNA in bladder tumor tissue is not common. International Urology and Nephrology 42, 95–102 (2010).
23) Zhang, L.et al. miR-937 contributes to the lung cancer cell proliferation by targeting INPP4B.Life Sciences 155,511110–115 (2016).
24) Zhang, X. et al. miR-589-5p inhibits MAP3K8 and suppresses CD90+ cancer stem cells in hepatocellular carcinoma. Journal of Experimental & Clinical Cancer Research 35, 176 (2016).
25) Gross, A. M.et al. Multi-tiered genomic analysis of head and neck cancer ties TP53 mutation to 3p loss. Nature513Genetics 46, 939–943 (2014).
26) Ge, Q. et al. The suppressive effects of miR-1180-5p on the proliferation and tumorigenicity of bladder cancer cells Histology and histopathology 32, 77–86 (2017).
27) Huang, J. et al. miR-92b targets DAB2IP to promote EMT in bladder cancer migration and invasion. Oncology Reports 36, 1693–1701 (2016).
28) Peng, F. et al. Identification of differentially expressed miRNAs in individual breast cancer patient and application in personalized medicine. Oncogenesis 5, e194 (2016).
29) Lei, L., Huang, Y. & Gong,W. Inhibition of miR-92b suppresses nonsmall cell lung cancer cells growth and motility by targeting RECK. Molecular and Cellular Biochemistry 387, 171–176 (2014).
30) Sun, Y. et al. Reduced miR-3127-5p expression promotes NSCLC proliferation/invasion and contributes to dasatinib sensitivity via the c-Abl/Ras/ERK pathway. Scientific Reports 4, 6527 (2014).
31) Fang, L., Yang, N.,Ma, J., Fu, Y. & Yang, G.-S. microRNA-1301-mediated inhibition of tumorigenesis. Oncology Reports 27, 929–934 (2012).
